# microRNAs targeting cellular cholesterol: implications for combating anticancer drug resistance

**DOI:** 10.18632/genesandcancer.202

**Published:** 2020

**Authors:** Bernice Monchusi, Mandeep Kaur

**Affiliations:** ^1^ School of Molecular and Cell Biology, University of Witwatersrand, Wits, Johannesburg, South Africa

**Keywords:** miRNAs, hsa-miR-128, hsa-miR-223, cholesterol, cancer drug resistance

## Abstract

Over sixty percent of all mammalian protein-coding genes are estimated to be regulated by microRNAs (miRNAs), and unsurprisingly miRNA dysregulation has been linked with cancer. Aberrant miRNA expression in cancer cells has been linked with tumourigenesis and drug resistance. In the past decade, increasing number of studies have demonstrated that cholesterol accumulation fuels tumour growth and contributes to drug resistance, therefore, miRNAs controlling cholesterol metabolism and homeostasis are obvious hypothetical targets for investigating their role in cholesterol-mediated drug resistance in cancer. In this review, we have collated published evidences to consolidate this hypothesis and have scrutinized it by utilizing computational tools to explore the role of miRNAs in cholesterol-mediated drug resistance in breast cancer cells. We found that hsa-miR-128 and hsa-miR-223 regulate genes mediating lipid signalling and cholesterol metabolism, cancer drug resistance and breast cancer genes. The analysis demonstrates that targeting these miRNAs in cancer cells presents an opportunity for developing new strategies to combat anticancer drug resistance.

## INTRODUCTION

miRNAs are small non-coding RNAs (20-22 nucleotides (nt)) [1] found to be present in animals and plants [2], of which some are conserved in bilaterian organisms.[3] For instance, almost 50 % of *Caenorhabditis elegans (C.elegans)* miRNA genes are reported to have homologs in humans [4]. According to the miRBase 22 release (http://www.mirbase.org), 38,589 entries represent hairpin precursor miRNAs, expressing 48,860 mature miRNA products in 271 species [5]. The first miRNAs, lin-4 and let-7 were found in *C. elegans* and both had imperfect complementary base-pairing with the 3’ untranslated region (UTR) of their target messenger RNAs (mRNAs) [6, 7]. It was later suggested by other studies that these regulatory RNAs or “small temporal RNAs” found in the worms were to regulate the timing of developmental changes [8]. The decoding of human genome has resulted in a surge of publications related to miRNAs. A simple PubMed search using keyword ‘microRNA OR miRNA’ (dated 04 May, 2020) have listed 107, 242 publications (one in 1972 and 15, 185 in 2019). This emphasizes miRNAs’ importance in modulating expression of genes involved in a large number of key signalling pathways as computational predictions of target mRNAs of all known miRNAs are shown to regulate > 60 % of all mammalian protein-coding genes [9]. Therefore, it is obvious to comprehend that deregulation of miRNAs will contribute to disease states and evidence have been gathered for diseases such as cancer and metabolic disorders [10, 11], autoimmune, cardiovascular and Alzheimer’s to name a few among plenty others [12]. Targeting miRNAs may therefore serve as a novel therapeutic intervention for treatment of various diseases.

A number of *in vivo* studies using oligonucleotides to block certain miRNA functions have shown efficacy in preclinical animal models [13]. The first miRNA therapeutic (Miravirsen) to block a human miRNA developed by Santaris Pharma entered a clinical trial in 2008 [14]. Miravirsen, an LNA-based (locked-nucleic acid) is an antisense molecule produced against miR-122 for the treatment of hepatitis C virus (HCV), and after successful safety evaluation in healthy volunteers, and initial trials in HCV patients [15], Miravirsen was proposed to undergo further larger scale trials. Recently Miravirsen was shown to specifically target mir-122 with no off target effects on other miRNAs in plasma levels of study patients [16]. Targeting miRNAs in cancer is also an emerging concept [17] as well as the role of miRNAs in cancer drug resistance has also been highlighted [18-20]. Therefore, in this review, we highlight the role of miRNAs in cancer and we have attempted to demonstrate the link of miRNAs with cancer drug resistance particularly through cholesterol-related pathways. For understanding this role of miRNAs, it is imperative to explore involvement of miRNAs in cancer, drug resistance and cholesterol related pathways. In the following sections, we explore these aspects of miRNA related biology and then we use an investigational approach to substantiate the role of miRNAs in cholesterol-mediated cancer drug resistance by using breast cancer as an example.

## RESULTS

### miRNA biogenesis

**Figure 1:**
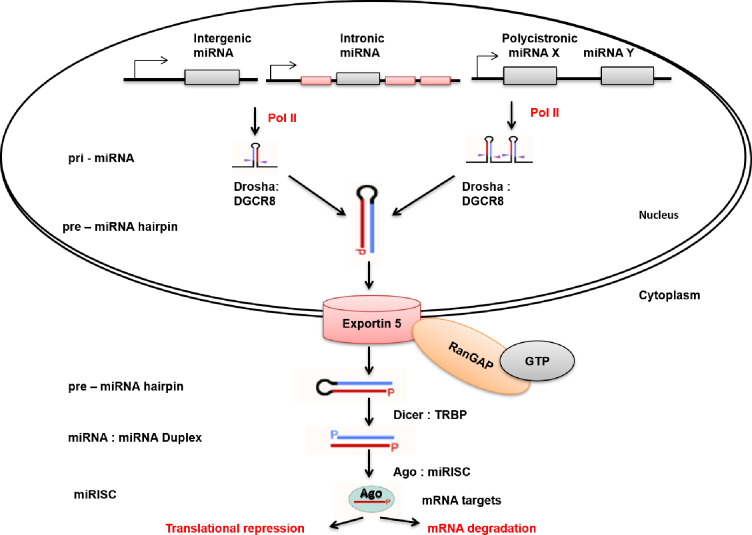
miRNA biogenesis pathway: Short intronic hairpins are the most common alternative miRNA biogenesis pathway, which are spliced and disbranched by Drosha: DGCR8 complex, to form pre-miRNA hairpins. The 2-nt 3’ overhang of the pre-miRNA is recognized by exportin 5: RanGAP forming a ternary complex in the nucleus. The ternary complex transports the pre-miRNA into the cytoplasm by diffusing through a nuclear pore complex. The hydrolysis of GTP to GDP by RanGAP results in a conformational change, releasing pre-miRNA into the cytoplasm. The loop region of the pre-miRNA is cleaved off by Dicer: TRBP resulting in the 22 nt mature miRNA: miRNA duplex. The miRNA duplex is incorporated into Ago-containing miRISC. In the miRISC, the mature miRNA is retained, directing the miRISC to complementary sites in the target mRNAs.

miRNAs are usually transcribed from intergenic, intronic or polycistronic loci into long primary transcripts called pri-miRNAs by RNA polymerase II (Figure 1) [21]. A hair-pin is formed by each pri-miRNA by folding back on itself, forming a substrate for the microprocessor. The microprocessor is a heterotrimeric complex that consists of two molecules of DGCR8 and one molecule of Drosha endonuclease [22] . In animals, pri-miRNA is usually transcribed by RNA polymerase II into mature miRNA through either the canonical or non-canonical miRNA biogenesis pathways. The canonical pri-miRNA is recognised and processed by the microprocessor (Drosha : DGCR8) into 70 nt pre-miRNA (precursor miRNA) by cutting one helical turn from the base of the hairpin [21]. Drosha consist of two RNase III domains that participate in the processing of the pri-miRNA hairpin [23]. The pre-miRNA 2-nt 3’ overhang is recognized by exportin 5 : RanGAP and is exported through the nuclear pore complex to the cytoplasm (Figure 1) [22] . The pre-miRNA is processed by a second RNase III enzyme, Dicer, into the miRNA duplex in the cytoplasm [24]. The miRNA duplex containing the miRNA paired to the messenger strand is generated when Dicer creates an incision near the loop region through interaction with the dsRBD protein transactivation response RNA binding protein (TRBP) [25]. Moreover, Dicer : TRBP recruits Argonaute (Ago) proteins to the miRNA-induced silencing complex (miRISC) to initiate assembly [24]. miRISC/RISC is a ribonucleoprotein complex that facilitates gene silencing by miRNA post-transcriptionally. Once the miRNA duplex is formed, it is loaded into a functional miRISC. In the miRISC the duplex unwinds and the mature miRNA strand is retained [22, 26]. The retained strand then partially forms complementarity with its target messenger RNA (mRNA) strand, leading either to translational repression or mRNA degradation. The choice of miRNA strand that is retained and becomes the guide strand depends on the orientation the duplex binds ago [22] . The binding pocket within Ago prefers the 5’-terminus, where it binds to the 5’-nucleoside monophosphate of the guide strand. The mature miRNA, still bound to the miRISC guides the silencing complex to target mRNAs through imperfect base pairing with their 3’ UTRs [27] .

**Table 1:** A non-exhaustive list of miRNAs aberrantly expressed in tumours.

miRNAs	Expression (Up/Downregulated)	Organ	Disease type	References
miR-21	Upregulated	Colon	Adenomas	[151]
MiR-200a-	Upregulated	Ovary	Carcinomas	[152]
miR-200c	Downregulated	Lung	NSCLC	[152, 154]
miR-1275	Upregulated	Pancreas	Cholangiocarcinomas	[3, 155, 156]
miR-29b, miR-21, -34a, -198, -217, 221, -483-3p, miR-125b, -145, -10b	Downregulated	Breast	Carcinomas	[157-159]

miRNA names are assigned according to a standard nomenclature system [28, 29]. The mature miRNAs are named using the ‘miR’ prefix in the primary database (www.mirbase.org/), whereas the pre-miRNA hairpins, as ‘mir’. The miR/mir prefix is followed by a dash and a unique identifying number, indicating the naming order (eg. miR-122 was named before miR-350) [29]. Species of origin is indicated with a three-letter prefix (eg, hsa in humans, dme in Drosophila melanogaster, and oar in sheep). In humans miR-122 will be labelled as hsa-miR-122. Distinct hairpin loci that result in mature miRNAs with 100 % identity are indicated as e.g. hsa-miR-224-1 and hsa-miR-224-2 in humans [28]. miRNAs with sequence identity differing by one or two nucleotides are indicated with an additional lowercase letter such as hsa-miR-224a and hsa-miR-224b. When two different miRNA sequences are cut from the same hairpin precursor, but at opposite arms, the sequences are named in the form hsa-miR-224-3p (3’ arm) and hsa-miR-224-5p (5’ arm).

Under normal physiological conditions, maintaining miRNA homeostasis is critical. However, in response to activation or stress, miRNA transcription, processing and functioning is rapidly altered. A quick search on PubMed using key words “miRNA dysregulation in cancer” (dated on 4th May 2020), a total of 2150 published articles came up. Unravelling the mechanisms of miRNA dysregulation in cancer could help in the discovery of new anticancer therapies.

### miRNAs in cancer

Historically, alterations in protein-coding genes (oncogenes or tumour suppressor genes) were thought to be the root cause of tumourigenesis [30, 31]. With the emergence of high-throughput methods, it came to light that non-coding RNA transcripts are produced by thousands of genes, with no significant open reading frames [32]. It is now well accepted that non-coding RNA transcripts such as miRNAs regulate genes that mediate development, cellular differentiation, hematopoiesis, stress tolerance, metabolism, cell proliferation, and apoptosis [33]. Several mechanisms of miRNA dysregulation in tumours have been experimentally proven including; point mutations in miRNA leading to decreased miRNA processing and rearrangement of the mRNA target 3’ UTR leading to suppression of miRNA expression [34]. The link between miRNA dysregulation and cancer was first presented by Calin et al., in 2002 [35]. It was reported that the clusters of the two miRNAs, miR-15 and miR-16 were often deleted in chronic lymphocytic leukemia (CLL), pointing out to the tumour suppressor activity of these miRNAs. Since then aberrant expression of miRNAs has been documented in several tumours [36, 37] (Table 1). Altered functioning of proteins participating in miRNA processing such as Dicer1, Ago2, Xpo 5 (encodes exportin 5) can also lead to aberrant expression of miRNAs. Abnormal expression levels of Drosha and Dicer have been found in various cancers including ovarian cancer [38].

Furthermore, tumours show resistance to DNA damage. Multiple pathways of DNA damage response (DDR) exist in mammalian cells to protect the genome by promoting apoptosis or by repairing the double-strand breaks. p53, also regarded as the “guardian” of the genome regulates the expression of DDR genes [39] and activates transcription of miRNAs along with the coding genes [40]. Among these miRNAs is the miR-34 family that act as tumour suppressors [41, 42]. On the other hand, p53 mutations lead to suppression of miR-34 in ovarian cancers leading to increase expression of MET (a cell-surface receptor tyrosine kinase encoded by Met gene) which in turn is involved in promotion of tumour cell proliferation, invasion and motility [43]. The abundance of miR-34 suppresses KRAS thus preventing tumour formation and progression[44]. Similarly, miR-24 modulates expression of histone H2A variant, H2AX, which represents double-stranded breaks and is involved in recruitment of DNA repair factors [45]. Interestingly, miR-24-mediated suppression of H2AX makes hematopoietic cells’ hypersensitive to gamma irradiation and genotoxic drugs, which leads to the reduction in the DNA repair capacity of terminally differentiated hematopoietic cells [46].

Hypoxia is a phenomenon that is common in tumours for survival and growth. The hypoxia-inducible factor 1 (HIF1) controls how these cells respond to hypoxic stress. It has been reported that hypoxia interferes with miRNA maturation by inhibiting the activity of Drosha and Dicer, promoting tumour progression [47]. miR-210 protects hypoxic tumour cells from apoptosis by modulating the expression of the pro-apoptotic Bcl-2 family member BNIP3 and the caspase-8-associated protein FLASH [48]. In addition, miRNAs can also either act as tumour promoters or suppressors of cancer initiation. miR-195, a tumour suppressor miRNA is reported to inhibit cell growth and enhances apoptosis after chemotherapy in hepatocellular carcinoma cells [49]. This effect is further found to be contributed by expression of cyclin D1, cyclin-dependent kinase CDK6, the transcription factors E2F3, and the pro-apoptotic protein BCL-2. Alternatively, miR-200 is frequently downregulated in human tumours resulting in EMT induction and leads to suppression of epithelial genes mediated by its targets ZEB transcription factors (ZEB1 and ZEB2), [50].

A plethora of literature has been published highlighting the involvement of several biochemical and signalling pathways in cancer development and progression. A less investigated cancer-related molecule is cholesterol and in recent years, it has been confirmed that numerous signal transduction pathways regulated by intracellular cholesterol are activated or dysregulated in cancer [51, 52]. When searching PubMed using key words “role of miRNAs in cholesterol dysregulation in cancer” (dated 4th May 2020), 3 articles came up which are not directly related to the key words used. It is therefore pertinent to establish whether there is a link between miRNAs in cholesterol dysregulation in cancer. The expression of 3-hydroxy-3-mehyl-glutaryl CoA (HMG-CoA) reductase enzyme, which is responsible for the rate limiting step of *de novo* cholesterol biosynthesis is elevated in cancer cells [53]. Previous experimental evidences have shown that cholesterol content in tumour cells is higher than that of non-cancerous cells [54]. A positive correlation between breast cancer risk and plasma cholesterol levels was found in breast cancer mice models [55]. Recently, high total serum cholesterol and high HDL was reported to be associated with increased risk to high-grade prostate cancer [56]. A positive association was found between high-density lipoprotein cholesterol (HDL-C) and apoA1 levels with increased breast cancer risk, 23 % and 28 % respectively [57].

Several miRNAs are reported to act at key steps during cholesterol biosynthesis and efflux to either initiate or repress tumorigenesis. miR-33a/b was shown to target ABCA1, an important regulator of HDL synthesis and reverse cholesterol transport. Moreover, inhibition of this miRNA upregulated ABCA1 expression and increased cholesterol efflux in mouse and human cell lines [58]. miR-128-2, a pro-apoptotic miRNA was shown to inhibit ABCA1, ABCG1 and RXRα mRNA and protein expression in cell lines such as MCF-7 breast cancer and HepG2 liver hepatocellular carcinoma [59]. Additionally, miR-223 was reported to directly repress scavenger receptor B1 thus regulating HDL-C uptake. Moreover, in human hepatocytes and macrophages, it also inhibits cholesterol biosynthesis through the direct repression of HMG-CoA synthase 1 and methylsterol monooxygenase 1 [60]. More recently, inhibition of cholesterol has been shown to reduce cell growth of breast cancer cells [61]. Cholesterol-lowering medication administered to patients with estrogen receptor positive (ER+) breast cancer during endocrine therapy has shown improved therapeutic outcomes (related to disease-free survival, breast cancer-free interval and distant recurrence-free interval) compared with nonusers [62].

From the above findings, it is clear that cholesterol homeostasis is important for normal cell function as altered expression could lead to disease state such as tumorigenesis. Therefore, it is of interest to further discuss and establish the role of miRNAs in cholesterol homeostasis to envision potentially new anticancer therapeutic strategies.

### Role of miRNAs in maintaining cellular cholesterol

**Figure 2:**
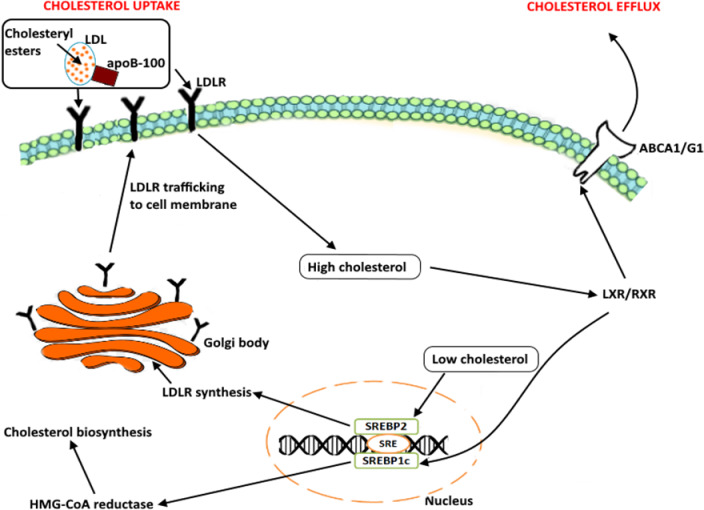
Cellular cholesterol regulation and homeostasis, Low cholesterol levels induce the expression of SREBPs (SREBP2 and SREBP1c). The activation of these SREBPs leads to the expression of cholesterol synthesis genes by binding to their sterol response element (SRE). SREBP2 promotes the synthesis of LDLR in the golgi apparatus, which is important for the uptake of cholesterol from the cell surface. SREBP1c induces the expression of HMG-CoA reductase that further promotes the biosynthesis of cholesterol. High cholesterol levels induce the expression of LXR/RXR, which leads to an increase in the expression of ATP-binding cassette transporters (ABCA1/ABCG1) leading to the efflux of free cholesterol from the cell.

Intracellular cholesterol levels are maintained through tightly regulated mechanisms, which include the endogenous biosynthesis, internalization of exogenous cholesterol and efflux of intracellular cholesterol (Figure 2). The transcription factors, Liver X receptors (LXRs) and sterol-regulatory element binding protein (SREBPs) regulate these mechanisms. The SREBPs influence the expression of genes that mediate cholesterol uptake and biosynthesis (Figure 2). While the LXRs/RXR mediate cholesterol efflux thereby maintaining cellular sterol homeostasis (Figure 2). Low cholesterol levels induce the expression of SREBPs (SREBP2 and SREBP1c), which upregulate the expression of HMG-CoA reductase and low-density-lipoprotein receptor (LDLR), leading to an increase in *de novo* biosynthesis and uptake of cholesterol, respectively (Figure 2) [52]. Excess cholesterol triggers a negative-feedback mechanism that is mediated by the LXRs (LXRa and LXRb).

Since the discovery of miR-122 as the first miRNA to regulate lipid metabolism, miRNAs have emerged as critical regulators of cholesterol homeostasis [63]. Further studies indicated that miR-122 and miR-370 upregulate SREBP-1c and genes involved in fatty acid and triglyceride biosynthesis [64]. miR-122 and miR-370 both target and regulate lipid metabolism by enhancing the expression of multiple coding genes, including, diacylglycerol O-acyltransferase 2 (DGAT2), fatty acid synthase (FAS), and acyl-CoA carboxylase (ACC1) [64-66]. Interestingly, ACC1 and FAS expression levels were depended on the levels of SREBP-1c and DGAT2 [64]. Furthermore, siRNA-based silencing of SREBP-1c or DGAT2 inhibited the miR-370- and miR-122-mediated upregulation of FAS and ACC1. Consistent evidence was found in another study showing that hyperlipidemia patients had significantly increased expression levels of miR-122 and miR-370 compared with controls [66]. This was further found to positively correlate with the total cholesterol, triglyceride and LDL-C (Low density lipoprotein cholesterol) in plasma levels of hyperlipidemia patients with coronary artery disease. For the first time in 2010, IIiopoulos et al. showed that miR-370 upregulates the expression of miR-122 and that the effect of miR-370 on upregulated lipogenic genes was greatly reduced by treatment with antisense miR-122 in HepG2 cells [64].

Cholesterol synthesis is regulated by several miRNAs e.g. miR-185 regulates the expression of HMG-CoA reductase and SREBP2 [67]. miR-128 as mentioned above regulates cholesterol accumulation and efflux by directly targeting the 3’ UTR of LDLR and ABCA1 [68], while miR-26a/b overexpression is reported to alter expression profiles of SREBP1 and LXRA [69]. Another miRNA, miR-758 is reported to also repress the expression of ABCA1 and conversely its inhibition by anti-miR-758 increased the expression of ABCA1 in mouse and human cells *in vitro*. In addition, miR-758 reduced cellular cholesterol efflux to apoA1, and anti-miR-758 increased it in mouse cells [70]. These observations suggest that targeting miRNAs that regulate cellular cholesterol may prove to be an attractive anticancer strategy. Additionally, it is also important to understand the role of miRNAs in modulating cancer-related drug resistance. Firstly, we will discuss the role of cholesterol in cancer drug resistance.

**Table 2:** miRNAs that play a role in cholesterol and cancer drug resistance.

miRNAS	Link to cholesterol	Cancer type	Mechanism	References
miR-106	Direct negative regulator of ABCA1	NSCLC carcinoma	Antisense inhibition of miR-106 increases effectiveness of anticancer drugs	[160, 161]
miR-34	Key regulator of hepatic homeostasis, by Suppressing SIRT1	Gastric cancer	Overexpression of miR-34 induces chemosensitization	[162, 163]
miR-122	Regulates genes that mediate hepatic cholesterol and lipid metabolism	Hepatocarcinoma	Increased expression of miR-122 sensitizes HCC cells to chemotherapeutic drugs	[164, 165]
miR-223	Negatively regulates cholesterol by inhibiting HMG-CoA synthase 1	Hepatocarcinoma	Overexpression of miR-233 increases sensitivity of HCC cells to anticancer drugs	[60, 166]
miR-128	Directly inactivates ABCA1, ABCG1, and RXRα expression	Breast tumours	Ectopic expression of miR-128 decreases ABCC5 and Bmi-1 while increasing chemosensitization of doxorubicin	[129, 136]
miR-33a	Downregulates TWIST, a novel ADD1/SREBP1c interacting protein expression	Osteosarcoma	Inhibition of miR-33a increased cell apoptosis and TWIST expression in Saos-2 cells.	[167, 168]

See accompanying text and references for more detailed information about mechanisms involved in the development of each cancer type.

### Role of cholesterol in cancer drug resistance

Approximately 40% of patients with resectable tumours and 80% unresectable tumours poorly respond to chemo- and radiotherapy [71] due to drug resistance. Chemoresistance can be intrinsic or acquired, occurring due to either lack of response to initial therapy (making therapy ineffective), or develops during treatment of tumours that were initially sensitive to the therapy, respectively [72]. Several mechanisms of drug resistance, that favour cancer cell’s survival have been reported in many studies including but not limited to; drug inactivation, drug efflux, drug target alteration, and DNA damage repair [73].

To date, a handful of studies have demonstrated experimental evidences that link intracellular cholesterol levels to cancer drug resistance. Cholesterol metabolism have been speculated to be involved in tamoxifen resistance in breast cancer cells [74], the standard endocrine therapy used to treat ER+ breast cancers [75]. A cholesterol depleting agent, methyl-β-cyclodextrin (MβCD) was found to suppress tamoxifen resistance, pro-survival signalling, and induced apoptosis when combined with tamoxifen in tamoxifen resistant breast cancer cells [76]. In another study, ER-positive breast cancer patients’ clinical outcomes were improved when they received cholesterol-lowering medication during their adjuvant endocrine therapy [62]. Furthermore, it has been shown that HMG-CoA reductase knockdown re-sensitized enzalutamide-resistant prostate cancer cells [77] to enzalutamide (a nonsteroidal second-generation antiandrogen). Recently, it was found that ALK+ anaplastic large cell lymphoma (ALCL) cells (a rare type of lymphoma) were unable to thrive in an environment that lacks cholesterol [78]. LDLR was identified as a necessity for the growth of ALCL cells using a CRISPR-based genetic screen and therapies blocking the uptake of cholesterol were considered to be effective against drug-resistant ALCL cells. Although opposite findings were reported [79], where intracellular cholesterol levels were lower in temozolomide (TMZ) resistant glioma cells compared to that of the control cells. TMZ is the known standard therapy for glioblastoma, however, continuous use was associated with an increased risk of developing resistance [79, 80].

The mechanism by which increased intracellular cholesterol confers cancer drug resistance is a topic that is currently gaining interest in cancer research. P-glycoprotein (P-gp)/ABCB1 which is encoded by the multidrug resistance gene (MDR1) is reported to be significantly upregulated post exposure to chemotherapy, in acquired drug resistance [81]. In another study, the expression of P-gp significantly increased in primary breast carcinomas after preoperative chemotherapy [82]. Similar findings was observed when breast cancers were treated by neoadjuvant therapy with fluorouracil, doxorubicin, and cyclophosphamide [83]. Interestingly, depletion of membrane cholesterol was shown to alter P-gp localization and abolish its efflux capability [84]. Similarly, altered expression of ABCA2 could increase uptake of LDL- derived free cholesterol thus increasing cellular cholesterol and was shown to be expressed with sterol-responsive genes [85]. This cellular cholesterol increase may further promote cancer chemoresistance. ABCA2 is primarily responsible for the transport of molecules such as lipids, cholesterol and pharmacological agents across cellular membranes. Overexpression of ABCA2 is associated with a variety of human pathologies and confers drug resistance phenotypes in several cancers including acute lymphoblastic leukemia (ALL) and lung cancer [86, 87]. Moreover, primary tumours from hepatocellular carcinoma (HC) patients exhibit increased mitochondrial cholesterol levels [88]. The increased cholesterol levels in HC cells imparted resistance to mitochondrial membrane permeabilization thus inhibiting release of cytochrome c in response to various stimuli such as active Bax. This effect was reversed upon cholesterol depletion by inhibition of hydromethylglutaryl-CoA (catalyses the first step of cholesterol biosynthesis) or squalene synthase (catalyses the final step of cholesterol biosynthesis) [88].

In recurrent prostate cancer (PCa), treatment usually entails androgen withdrawal therapy using androgen receptor (AR) antagonists leading to suppression of androgen production [89]. Castration resistant PCa (having the ability to continuously produce androgens despite castration) usually develops in patients who initially responded to such treatment [90]. Increase in cholesterol levels may promote castrate resistant PCa as these cells were able to synthesise androgens from cholesterol precursors [91]. Roy et al. proposed the suppression of androgen production to be one of statins (cholesterol inhibitor) mechanism of action [92] as cholesterol is a required intermediate in androgen synthesis in the testis and adrenal glands [93]. MBCD (methyl-β-cyclodextrin), another cholesterol depleting agent was shown to induce apoptosis through decreasing raft levels, Bcl-xL downregulation, caspase-3 activation and Akt inactivation in A431 human tumour cells [94]. Alternatively, cholesterol repletion restored Akt activation and cell viability by replenishing rafts on the cell surface. Furthermore, the breast cancer and prostate cancer cell lines contained increased lipid rafts and demonstrated more sensitivity to cholesterol depletion-induced cell death than normal cells.

Based on these findings, it is clear that the role of cholesterol in cancer drug resistance is not completely understood. It is therefore pertinent to explore the role of emerging miRNAs, as a mechanism in cholesterol-mediated cancer drug resistance in different cancers.

### Roles of miRNAs in cholesterol-mediated drug resistance

Several studies have implicated aberrant miRNA expression with anticancer drug resistance in many cancers including breast cancer [95]. Some miRNAs such as miR-125b, miR-181a, and miR-126-5p have been shown to be upregulated in drug resistance [96-98], while other miRNAs such as miR-103/107 are downregulated [99]. These observations suggest an important biological role of miRNAs in drug resistance however, their role in cholesterol-mediated cancer drug resistance is at its very beginning. Few studies have reported on miRNAs that regulate cholesterol, as potential anticancer drug targets for combating cancer drug resistance (Table 2).

Additionally, miR-301a has been linked with drug resistance of osteosarcomas (OS), where over-expression of miR-301a correlated with reduced doxorubicin-induced apoptosis [100]. It was observed that doxorubicin increased expression of HMG-CoA reductase while inhibiting AMPKα1 expression in OS cells, which was further supported by over-expression of miR-301a in OS cells. Conversely, anti-miR-301a enhanced apoptosis in OS cells. Consistently, miR-301a and HMG-CoA reductase were upregulated in chemoresistant OS cells compared to control OS cells. Overexpression of miR-181a sensitized mitoxantrone (MX)-resistant MCF-7 cells by downregulating ABCG2/BCRP expression [101]. ABCG2 recognizes and transports a variety of chemotherapeutic drugs out of cancer cells, reducing drug concentration and resulting in drug resistance [102]. *In vivo*, intratumoral injection of miR-181a mimic inhibited ABCG2 expression and enhanced the anti-tumour activity of MX in a nude mouse xenograft model [103]. ABCG2 has been found to be located in membrane rafts and cholesterol was reported to have an impact on its efflux activity [103]. Cholesterol depletion with MβCD significantly decreased the activity of ABCG2 by 40%. Moreover, cholesterol repletion with cholesterol-inclusion complexes restored ABCG2 function. Moreover, miR-195 overexpression was reported to significantly alter cellular cholesterol and triglyceride levels which further translated into reduced proliferation, invasion and migration of MCF-7 and MDA-MB-231 cells [104]. Singh et al. for the first time reported ACACA, FASN and HMG-CoA reductase as direct targets of miR-195 [105]. Lastly, miR-185 and miR-342 have been shown to control lipogenesis and cholesterogenesis by inhibiting SREBP-1 and -2 expression and downregulating their target genes (FASN and HMG-CoA reductase) in prostate cancer [105]. Furthermore, it was demonstrated that miR-185 and miR-342 overexpression inhibits cell growth, tumourigenicity, migration and invasion of prostate cancer cells and in xenograft models which coincided with the inhibited lipogenesis and cholesterogenesis.

There are only a handful of evidences reported on the role of miRNAs in cholesterol-mediated drug resistance. It is known that cholesterol content of cancer cells is higher than that of non-cancerous cells [54] and evidences are emerging that cholesterol accumulation is directly associated with cancer drug resistance, as discussed above [74]. It is therefore pertinent to investigate this further and in the following section we have attempted to explore miRNA targets of two miRNAs involved in cholesterol-mediated drug resistance. We anticipate that this investigation will open-up a new direction of research and will also add on to existing literature. hsa-miR-128 and hsa-miR-223 were selected for further analysis as these miRNAs regulate cholesterol genes that mediate cholesterol export and biosynthesis, respectively [68, 106]. Breast cancer was selected as it is the most prevalent female cancer worldwide [107], and breast cancer cells are also confirmed to be rich in cholesterol [54].

### hsa-miR-128 and hsa-miR-223 potentially regulate genes involved in cholesterol-mediated drug resistance in breast cancer

We provide insight into potential targets of hsa-miR-128 and hsa-miR-223, to explore their role in cholesterol-mediated drug resistance using bioinformatics tools. Gene lists containing 84 genes involved in three biological pathways (RT^2^ Profiler™ PCR Array Human Cancer Drug Resistance: PAHS-004Z, RT^2^ Profiler™ PCR Array Human Lipoprotein Signaling & Cholesterol Metabolism: PAHS-080Z and RT^2^ Profiler™ PCR Array Human Breast Cancer: PAHS-131Z) were obtained from Qiagen (https://www.qiagen.com). To identify hsa-miR-128 and hsa-miR-223 target genes, OmicsNet (http://www.omicsnet.ca/faces/home.xhtml) was used. The networks generated included hsa-miR-128-3p, hsa-miR-223-3p, and hsa-miR-223-5p with their corresponding genes. However, the -5p arm of hsa-miR-128 was not available on OmicsNet. Under the filter section, the gene lists obtained above were inserted and genes that were present in the networks were highlighted. Special filter functions were then employed to extract the miRNAs with the highlighted genes [108].

**Figure 3:**
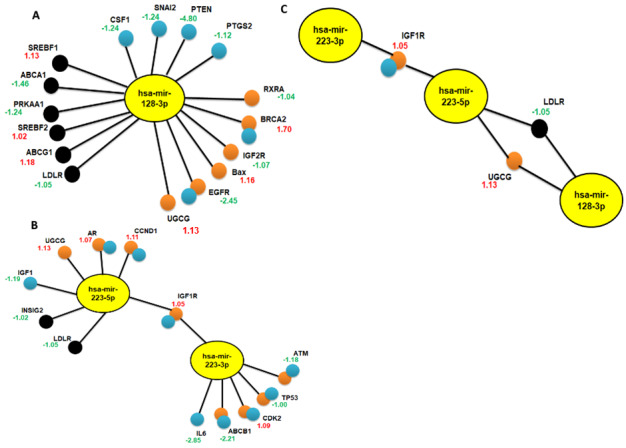
Networks depicting genes regulated by hsa-miR-128 and hsa-miR-223, respectively. OmicsNet was used to construct microRNA-gene interaction networks between hsa-miR-128, hsa-miR-223 and their target genes. The networks represent genes that correlated with the query genes, regulated by hsa-miR-128-3p **A**, and hsa-miR-223-3p: has-miR-223-5p **B.** **C**. The 3^rd^ network represents genes that are regulated by both miRNAs (hsa-miR-128 and hsa-miR-223 (3p/5p)). Genes involved in the following pathways were depicted as; orange circles: cancer drug resistance, black circles: lipoprotein and cholesterol metabolism, and blue circles: breast cancer. The edges that connect neighbouring genes are depicted as bold black lines. GEPIA (http://gepia.cancer-pku.cn/) was used for differential gene analyses of breast tumour tissue samples relative to normal tissues of hsa-miR-128 and hsa-miR-223 target genes. Increase in fold change of expression (upregulated genes) is represented as values in red colour and decrease in expression (downregulated genes) in green.

From the 84 cancer drug resistance genes used as input, six genes (RXRA, BRCA2, IGF2R, BAX, EGFR, and UGCG) were found to be regulated by hsa-miR-128-3p, while eight genes (AR, UGCG, CCND1, IGF1R, ATM, CDK2, TP53, and ABCB1) were regulated by hsa-miR-223-3p and hsa-miR-223-5p (Figure 3A and 3B). When the lipoprotein and cholesterol metabolism genes (84 genes) were used as input, six genes (SREBF1, ABCA1, PRKAA1, SREBF2, ABCG1, and LDLR) were found to be regulated by hsa-miR-128-3p while only two genes (INSIG2, and LDLR) were regulated my hsa-miR-223-5p (Figure 3A and 3B). Lastly, from the 84 breast cancer genes used, six genes (CSF1, SNAI2, PTGS2, PTEN, BRCA2, and EGFR) were found to be regulated by hsa-miR-128-3p while nine genes (AR, ATM, CCND1, CDK2, IGF1, IGF1R, TP53, IL6, and ABCB1) were regulated by hsa-miR-223-3p and hsa-miR-223-5p (Figure 3A and 3B). Interestingly, two genes (LDLR and UGCG) were regulated by both, hsa-miR-128-3p and hsa-miR-223-5p (Figure 3C). Cells obtain cholesterol from the LDLRs present on the cell surface [109]. High expression of LDLR has been associated with tumours from breast cancer cells in mouse models with hyperlipidemia [110]. Furthermore, knockdown of LDLR was found to reduce tumour growth in these mouse models. UDP-glucose ceramide glucosyltransferase (UGCG) is a known key enzyme involved in the synthesis of glycosylated sphingolipids. Recently, overexpression of UGCG has been shown to enhance proliferation and doxorubicin resistance in MCF-7 cells [111].

Increased cholesterol uptake and biosynthesis seems to stimulate tumour growth, which may result in drug resistance. It has been found that in drug-resistant colon cancer HT29-dx cells, cholesterol content was higher compared to the drug-sensitive HT29 cells, which was decreased post-treatment with MβCD, a cholesterol-lowering agent [112].

Interestingly, both miRNAs were found to regulate the insulin-like growth factor receptors (hsa-miR-128-3p: IGF2R and hsa-miR-223 (3p and 5p): IGF1R (Figure 3A and 3B). These IGF receptors are important regulators of cell growth and survival. The IGF-IGF1R axis includes the IGF1R, IGF2R, and insulin receptor (INSR) [113]. IGF-1, IGF-2 and serum insulin-like growth factor binding proteins (IGFBPs) are ligands known to bind to these receptors. It has been reported that the IGF- IGF1R axis receptors are overexpressed in malignant tumours [114]. IGF1R overexpression has been reported in patients with gastric cancer [115]. In another study, a decrease in IGF1R expression has been associated with an improved chemotherapy response in patients with human EGFR2 negative breast cancer [116]. The role of IGF1R in cholesterol biosynthesis and homeostasis is largely unknown. Interestingly, the IGF1 ligand was also shown to be regulated by hsa-miR-223-5p (Figure 3B). Previously, four hours treatment of murine C2C12 myoblasts cells with IGF1 has been shown to induce five fatty acid genes and nineteen cholesterol biosynthesis genes [117]. LDLR (2-fold change) that mediates uptake of cholesterol was amongst the cholesterol genes that were upregulated. Increase in IGF2R expression was reported in 198 patients with NSCLC while 266 had low expression of IGF2R [118]. It was shown that patients with low IGF2R expression had poorer prognosis after chemotherapy. Cholesterol amongst other ligands is known to bind IGF2R for delivery of Poly (rC)-Binding Protein 2 (PCBP2) siRNA to hepatic stellate cells (HSCs) [119]. These findings suggest that selected miRNAs (miR-128 and miR-223) may be potential anti-cancer drug resistance targets. These findings were further validated by assessing breast cancer tumour samples and breast cancer survival analysis plots.

### hsa-miR-128 and hsa-miR-223 target genes in breast cancer

Gepia (http://gepia.cancer-pku.cn/) was used for differential expression analyses of hsa-miR-128 and hsa-miR-223 target genes in breast tumour tissue and normal samples. GEPIA consists of RNA sequencing expression data of 9, 736 tumours and 8, 587 normal samples from the Cancer Genome Atlas (TCGA) and the Genotype Tissue Expression (GTEx) projects [120]. Altered expression was found in all target genes of hsa-miR-128 and hsa-miR-223 compared to the normal tissue samples, which mediate breast cancer, drug resistance and lipoprotein and cholesterol signalling (Figure 3). Amongst other genes upregulated in breast tumour tissue was UGCG (1.13-fold change), regulated by both hsa-miR-128 and hsa-miR-223, which is involved in drug resistance. Consistent with these observations, levels of UGCG (*p* = 1.5x10^-4^, fold change = 2.953) were higher in mixed lobular and ductal breast carcinoma tumours compared to normal breast tissue as analysed by Oncomine database (https://www.oncomine.org/resource/main.html) [111, 121] using TCGA breast dataset (Supplementary Figure 1A). Interestingly, SREBF1 (1.13-fold change), a transcription factor mediating cholesterol biosynthesis, regulated by hsa-miR-128, expression levels were also higher in breast tumours (*p* = 0.008, fold change = 2. 364) as compared to normal breast tissues analysed by Oncomine database (Supplementary Figure 1B). It is also noteworthy that from the bar chart view, selecting UGCG or SREBF1 as potential biomarkers would be unlikely as most normal breast tumour samples also expressed high levels of these genes (Supplementary Figure 2A-2B). On the contrary, SREBF2 (1.02-fold change) was found to be highly expressed in breast tumours analysed by GEPIA, however, was found to be relatively under expressed as analysed by Oncomine. Only a small subset of mucinous breast carcinomas tumours was observed to have high expression of SREBF2 (*p* = 0.002, fold change = 1,229) (Supplementary Figure 1C). RXRA (-1.04 fold change) was also under expressed in breast tumours as compared to normal samples, consistent with analyses by Oncomine database (Supplementary Figure 1F). However, RXRA (*p* = 0.007, fold change = 1.552) was only highly expressed in mixed lobular and ductal carcinoma tumours. LDLR (-1.05-fold change), which is crucial for cholesterol transportation, regulated by both has-miR-128 and has-miR-223, was downregulated in breast tumour samples as compared with normal samples analysed by both, GEPIA (Figure 3A-3B) and Oncomine database (Supplementary Figure 1E).

Interestingly, the expression levels of PTGS2 (-4.80-fold change) and EGFR (-2.45-fold change) regulated by hsa-mirR128, ABCB1 (-2.21-fold change), IGF1 (-2.19-fold change), and IL6 (-2.85-fold change) regulated by hsa-miR-223, were significantly reduced in breast tumour tissue. These results were consistent with analyses by Oncomine database, except for IGF1 whose expression was high in all breast tumour samples (Supplementary Figure 1J). High levels of IGF1 has been correlated with poor prognosis in patients undergoing endocrine therapy [122]. Quick literature search revealed that PTGS2, EGFR, ABCB1, and IL6 high expression rather than low expression was correlated with breast cancer growth. Prostaglandin endoperoxide synthase 2 /cyclooxygenase-2 (PTGS2) is important in regulating inflammatory responses [123]. Expression of PTGS2 has been suggested to promote breast cancer growth **in vitro** [124]. Epidermal growth factor receptor (EGFR) is altered in triple-negative breast cancer (TNBC), however, there are currently no EGFR targeting therapies approved for the treatment of breast cancer [125]. ATP-binding cassette sub-family B member 1 (ABCB1) was found to be upregulated (47 fold) in docetaxel-resistant MCF-7 and MDA-MB 231 cell lines [126]. According to data analysed by Oncomine, (Supplementary Figure 1I, 1K, and 1L) mucinous breast carcinoma was the only breast tumour that had high expression of PTGS2, EGFR, and ABCB1, suggesting that expression of these genes vary based on breast cancer type. Moreover, Interleukin-6 (IL6) function has been reported to be important in the growth and metastasis of breast cancer cells and drug resistance of breast cancer stem cells [127]. These findings suggest that hsa-miR-128 and hsa-miR-223 may be potential targets to combat cholesterol-mediated drug resistance in breast cancer.

Based on these findings and our bioinformatics analyses it can be inferred that an increase in cholesterol uptake and accumulation related genes may promote breast cancer development. We therefore hypothesise that targeting either hsa-miR-223 or hsa-miR-128 directly or indirectly through their targets could be an attractive anticancer therapy.

**Figure 4:**
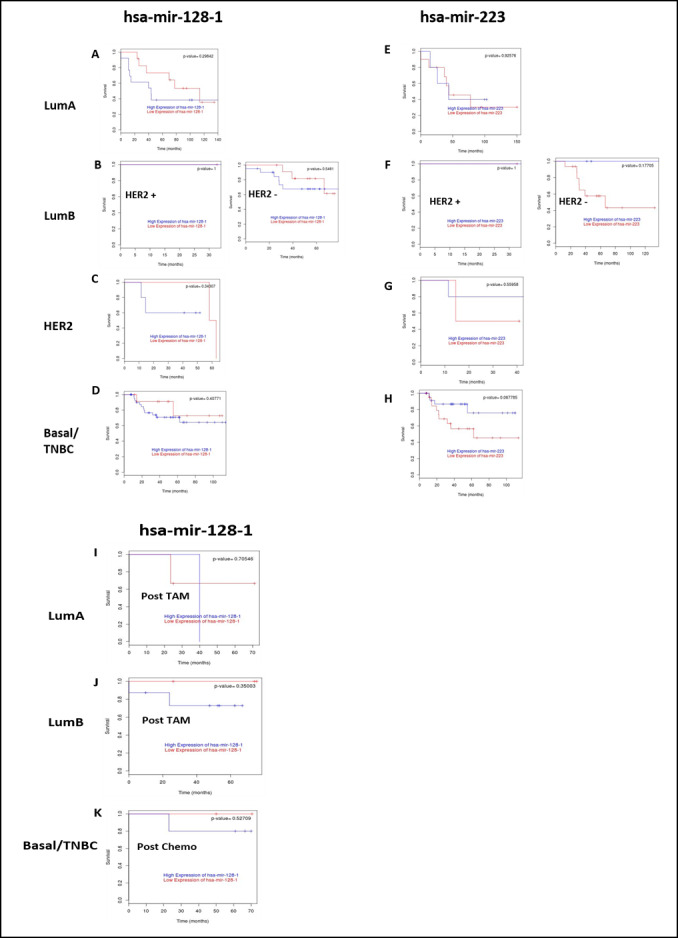
Kaplan-Meier plots depicting patient survival before and post-treatment in three breast cancer subtypes. Prognostic effect of hsa-miR-128-1 **A**-**D**. and hsa-miR-223 **E**-**H** expression in breast cancer patients of different subtypes was assessed using MTCI breast cancer survival analysis tool (http://glados.ucd.ie/BreastMark/). The plots represent Disease-free survival (DFS) Kaplan-Meier plots constructed with MTCI. The red lines represent survival data in breast cancer patients where the queried miRNA is significantly below the median, while the blue lines represent those where the queried miRNA was significantly above the median. **A**-**D**. Effects of hsa-miR-128 expression on LumA: *n* = 25, number of events = 14, *p* = 0.298; LumB (HER+): *n* = 2, number of events = 0, *p* = 1; LumB: *n* = 33, number of events = 9, *p* = 0.548; Basal/TNBC: *n* = 55, number of events = 14, *p* = 0.407. **E**-**H**. Effects of hsa-mir-223 expression on LumA: *n* = 15, number of events = 9, *p* = 0.925; LumB (HER2+): *n* = 2, number of events = 0, *p* = 1; LumB(HER2-): *n* = 19, number of events = 7, *p* = 0.177; Her2: *n* = 7, number of events = 4, *p* = 0.559; Basal/TNBC: *n* = 47, number of events = 13, *p* = 0.067. **I**-**K**. The effects of tamoxifen (TAM)/chemotherapy (Chemo) post-treatment were assessed on grade 3 breast cancer patients with hsa-miR-128-1 expression. **I**-**J**. Effects of tamoxifen on hormone responsive breast cancer patients (LamA: *n* = 4, number of events = 2, *p* = 0.706; LumB: *n* = 11, number of events = 2, *p* = 0.350), and **K** effects of chemotherapy on TNBC patients (*n* = 7, number of events = 1, and *p* = 0.527) expressing hsa-miR-128-1.

### Breast cancer survival analysis

To further confirm the effects of hsa-miR-128 and hsa-miR-223 expression on the prognosis of breast cancer patients, the MTCI Breast Cancer Survival Analysis Tool (http://glados.ucd.ie/BreastMark/) was used to construct disease-free survival (DFS) Kaplan-Meier plots. MCTI integrates gene expression and survival data from 26 datasets on 12 different microarray platforms (~17,000 genes in up to 4,738 samples) [128]. ER+ and TNBC was of interest as there are currently no target therapies available for TNBC and drug resistance is a major problem in patients with ER+ breast cancer. High expression of hsa-miR-128-1 was associated with poor DFS in all grade 3 breast cancer patients irrespective of subtypes (Figure 4A-4D). However, in the setting of HER2 subtype (Figure 4C) even though low expression of hsa-miR-128-1 was associated with patient survival, worse DFS is observed after 50+ months. Low expression of hsa-miR-223 were inversely correlated with survival in grade 3 breast cancer patients irrespective of subtypes (Figure 4E-4H). While, high expression of hsa-miR-223 was associated with patient survival. No effect of hsa-miR-128-1 (Figure 4B) and hsa-miR-223 (Figure 4F) expression was seen on patient survival of luminal B (HER2+) subtype, as DFS remained constant.

Based on these findings, it can be postulated that lowering hsa-miR-128-1 expression or increasing the expression of hsa-miR-223 would increase survival of breast cancer patients. Effects of tamoxifen/chemotherapy on patients’ survival were further assessed. No data were available post tamoxifen/chemotherapy treatment in breast cancer patients of all subtypes with hsa-miR-223 expression in MTCI database. Worse DFS was observed in breast cancer patients of LumA subtype with high expression of hsa-miR-128-1 post tamoxifen treatment, while an improvement in DFS was evident in breast cancer patients with low expression of has-miR-128-1 (Figure 4I). Similarly, LumB subtype patients expressing low levels of hsa-miR-128-1 showed improved DFS, however, no significant improvement in DFS was evident in patients expressing high levels of hsa-miR-128-1 (Figure 4J). These findings reinforce the importance of decreasing the expression of hsa-miR-128-1 during endocrine therapy for better DFS outcomes of breast cancer patients. Furthermore, a significant improvement in DFS of TNBC patients expressing low levels of hsa-miR-128-1 was observed post chemotherapy (Figure 4K). It is to be noted here that the observed differences in DFS based on high and low expression of queried miRNA were not statistically significant (as evident from p-values), but this may be due to the limited number of patients that were selected by the database to match the query.

### Proposed model for targeting cholesterol for miRNA-mediated therapeutics

**Figure 5:**
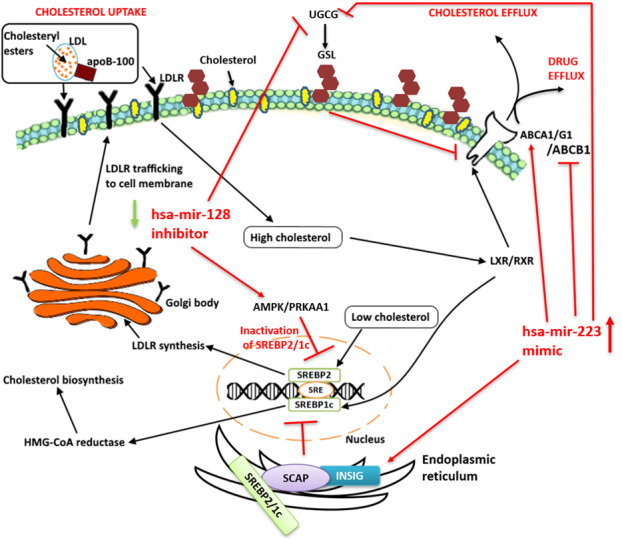
Hypothetical model (in red text and lines) presenting ways in which cellular cholesterol could be lowered in breast cancer patients by targeting hsa-miR-128 and hsa-miR-223. Bioinformatics analysis using the latest version of OmicsNet, accessed on 18 July 2019 revealed SREBP2, SREBP1c, AMPK, ABCA1, ABCG1, RXRa, and UGCG as targets of hsa-miR-128. Here we propose that, using anti-miR oligonucleotides of hsa-miR-128, can decrease the expression of SREBP2 while increasing the expression of AMPK, thereby leading to low cellular cholesterol. Moreover, inhibiting hsa-miR-128 could lead to a decrease in the expression of UGCG, a drug resistance gene. OmicsNet also revealed INSIG2 and UGCG as targets of hsa-miR-223 and interaction between SCAP and INSIG1 or INSIG2 keeps the SCAP/SREBP complex in the endoplasmic reticulum. We propose that increasing the expression of hsa-miR-223 (using a mimic) could directly increase the expression of INSIG, thereby keeping SCAP from undergoing conformational change to assist in the transportation of SREBP, which mediates cholesterol synthesis. Increasing the expression of has-miR-223 could also lead to the inhibition of UGCG thereby promoting cholesterol efflux through ABCA1. Furthermore, increasing the expression of hsa-miR-223 may decrease the expression of ABCB1 indirectly, leading to drug efficacy.

To put above discussion and insights into perspective, we propose a hypothetical model for reducing cholesterol-mediated drug resistance by either hsa-miR-128 or hsa-miR-223 in breast cancer (Figure 5). Adlakha and colleagues proposed that low levels of intracellular cholesterol induced SREBP2 expression, which may in turn induce the expression of hsa-miR-128 (Figure 45) [129]. This was further supported with bioinformatics-based analysis by TRANSFAC search which revealed the presence of SREBP transcription factor binding site in the promoter region of ARPP21 (CAMP regulated phosphoprotein 21) gene, the gene that encodes miR-128-2. Once induced, hsa-miR-128-2 in a positive feedback loop, increases the expression of SREBP2 leading to the synthesis and accumulation of cellular cholesterol, a phenomenon which is evident in most cancer cells. Adlakha and colleagues further showed through *in vitro* studies that miR-128-2 increases the expression of SREBP2 and decreases the expression of SREBP1 in HepG2, MCF-7 and HEK293T cells independent of sirtuin 1 (SIRT1) status [59]. These findings suggest that miR-128-2 increases cholesterol uptake by promoting LDLR synthesis via SREBP2 while decreasing cholesterol biosynthesis probably by inhibiting the induction of HMG-CoA reductase *via* SREBP1. They further found that overexpression of miR-128-2 inhibited the expression of ABCA1, ABCG1, and RXRα directly through a miR-128-2 binding site within their respective 3’ UTRs. Moreover, cholesterol efflux was inhibited by overexpression of miR-128-2, while miR-128-2 silencing stimulated cholesterol efflux in mice fed a high fat diet. Additionally, ovarian cancer mutant cells/SREBF2-KD (SREBF2 disrupted using CRISPR technology) treated with paclitaxel in low, but not high serum or in presence of statin showed a significantly lower cell viability [130]. In another study, SREBP2 mRNA expression was found to be increased in A2780-resistant ovarian cancer cell line using bioinformatics analysis [131].

Conversely, their previous study revealed that miR-128-2 increased the antitumor effect of compounds that target the p53 pathway [132]. In this study they found that overexpression of miR-128-2 induced apoptosis in a p53-dependent and -independent manner via the induction of PUMA in HEK293T and MDA-MB 231 cells. This is further supported by a study by Hu et al. in which they found that miR-128 was significantly downregulated in non-small cell lung cancer (NSCLC) tissues and cancer cells [133] . Furthermore, that the *In vivo* restoration of miR-128 significantly suppressed tumourigenicity of A549 cells in nude mice and inhibited both angiogenesis and lymphangiogenesis of tumour xenografts. Recently, in another study miR-128-3p along with miR-33a-5p were expressed at low levels in whole blood of lung cancer patients or early-stage lung cancer patients (TNM stage I-II) as compared with that in healthy controls [134]. Although contrasting results have been obtained so far, but it is to be noted that low levels of miR-128-3p are present in early stages of lung cancer, whereas overexpression of this miRNA has been linked with activation of β-catenin and TGF-β signalling, leading to metastasis and chemoresistance, which were reduced by antagonizing miR-128-3p [135]. In a previous study, ectopic expression of hsa-miR-128 (not specified whether -3p or -5p) was found to sensitise chemoresistant breast tumour-initiating cells (BT-ICs) to the proapoptotic and DNA-damaging effects of doxorubicin [136]. Therefore, it can be inferred from our predictive analysis that SREBP2 could be inhibited by targeting hsa-miR-128-3p in breast cancer and that silencing miR-128-3p as an adjuvant could increase the effectiveness of endocrine therapy thereby possibly eliminating drug resistance, although this can be dependent on the stage of the cancer.

Furthermore, SREBP2 is known to be inactivated by AMP- activated protein kinase (AMPK, also known as PRKAA1). It has been shown that increased proteolytic processing of SREBP1c and SREBP2 is prevented by activation of AMPK in the liver of insulin-resistant mice [137]. Based on the analysis shown earlier (Figure 3A), we hereby propose that hsa-miR-128-3p increases the expression of SREBP2 by decreasing the expression of AMPK. Inhibiting hsa-miR-128-3p with anti-miR-128-3p inhibitors could result in a decrease in the expression of SREBP2 in breast cancer by preventing inactivation of AMPK (Figure 5).

Moreover, we also propose that overexpressing hsa-miR-223 with oligonucleotides that mimic its expression could have different effects on the cell. In sterol-rich environment, the INSIG proteins (INSIG1 and INSIG2) bind the sterol regulatory element-binding protein cleavage-activating protein (SCAP) in the endoplasmic reticulum, thereby preventing the release of SCAP-SREBP complex [138]. The SCAP-SREBP complex is important for proteolytic processing of SREBP1a, SREBP1c, and SREBP2 isoforms into active transcription factors, which regulate expression of several cholesterol pathway related genes. The SCAP-INSIG ratio in the cell is crucial for sterol sensing to regulate SREBP processing, thus altering cholesterol synthesis [139]. On the contrary, INSIG2 has been found to be overexpressed in colorectal cancer tissue where it contributes to poor survival and promotes malignant behaviour [140] as cellular proliferation, invasion, and anchorage-independent growth increased while apoptosis reduced. In a previous study by Kayashima et al. they showed that all pancreatic cell lines including PANC-1 and MIA PaCa-2 pancreatic cells expressed INSIG2 mRNA [141]. With the latter pancreatic cells expressing > 2-fold higher INSIG2 mRNA expression levels under hypoxic conditions (1% O_2_) as compared to under normoxic conditions (21% O_2_). They found that cell proliferation and invasion was significantly decreased in SUIT-2 cells after transfection with INSIG2-targeting siRNAs. To date no direct correlation has been established between INSIG2 expression and breast cancer. Therefore, we speculate that INSIG2, a negative regulator of cholesterol uptake and biosynthesis might possibly when overexpressed in breast cancer by miR-223-5p increases effectiveness of endocrine therapy and eliminate drug resistance.

Moreover, plasma membrane lipid rafts are known to be mainly composed of cholesterol and glycosphingolipids (GSL). Increased levels of cholesterol have been reported to mask membrane GSLs in human tumour biopsies making immunotherapy ineffective [142, 143]. In a previous study, GSL accumulation has been found to inhibit cholesterol efflux through the ABCA1 pathway [144]. Since hsa-miR-223 has been found to positively regulate ABCA1 through its direct target Sp3 transcription factor to increase cholesterol efflux [145], we hereby suggest that overexpression of hsa-miR-223 could prevent cholesterol accumulation by increasing ABCA1 expression and by inhibiting UGCG, a drug resistance gene that catalyses the first glycosylation step in GSL biosynthesis (Figure 5). Lv et al. found miR-760 to be significantly downregulated in chemoresistant breast cancer tissues as compared to chemosensitive tissues [146]. They further predicted three target genes including ABCA1 to be involved in chemoresistance of MCF-7 cells to doxorubicin. Conversely, exposure of H1299 lung cancer cells to escalating doses of α-Tocopheryl succinate made these cells resistant to the agent due to the upregulation of the ABCA1 protein, which caused its efflux [147]. Additionally, overexpression of UGCG in MCF-7 breast cancer cells enhanced proliferation and promoted doxorubicin resistance [148]. These findings iterate the importance of possibly overexpressing miR-223 in breast cancer. In a previous study, mutant p53 was reported to reduce hsa-miR-223 expression, leading to upregulation of Stathmin 1 (STMN1) and increased chemoresistance in breast cancer cell lines [149]. Based on these findings, in the current study we anticipate that increasing the expression of hsa-miR-223 could upregulate the expression of ABCA1 while inhibiting the expression of UGCG, thereby increasing cholesterol efflux, thus sensitizing ER+ breast cancer resistant cells or TNBC cells to chemotherapeutic agents.

We also speculate that hsa-miR-128 when expressed, increases the expression of UGCG, it is therefore pertinent to reduce the expression of hsa-miR-128. Moreover, hsa-miR-223 could further increase drug efficacy by downregulating ABCB1/MDR1, a well characterized ABC-transporter. Besides hsa-miR-33a [150], hsa-miR-223 and hsa-miR-128 seem to be new components joining the SREBP signalling pathway. Based on the above observations, targeting either hsa-miR-128 or has-miR-223 seemed to be important in preventing cholesterol-mediated drug resistance.

## FUTURE PERSPECTIVES AND CONCLUSIONS

Presently, cancer drug resistance remains a problem that needs to be addressed. miRNAs have been shown to regulate genes from numerous biological processes, including cholesterol metabolism and cancer. Recently, miRNAs have been implicated in cancer drug resistance. However, little is known about the role of miRNAs that regulate cholesterol-mediated drug resistance in breast cancer. In the present review, we explored the possibility of miRNAs to be involved in cholesterol-mediated cancer drug resistance. Our bioinformatics analysis gives some insight into this concept. Targeting miRNAs (hsa-miR-128 or hsa-miR-223) may provide an opportunity for anti-cancer drug discovery and development. We find that hsa-miR-128 and hsa-miR-223 regulate genes mediating lipid signalling and cholesterol metabolism, as well as cancer drug resistance in breast cancer. We propose that either inhibiting hsa-miR-128 or increasing hsa-miR-223 expression could modulate the expression of cholesterol pathway related genes thereby lowering cellular cholesterol. Through this mechanism, we speculate that these miRNAs may be involved in cholesterol-mediated cancer drug resistance. To date, experimental evidence is lacking to confirm this hypothesis, nonetheless, the computational analysis provides an avenue that needs to be further explored experimentally, although caution must be practised in light of the fact that miRNA target genes may contribute to several downstream pathways (e.g. INSIG2 as discussed above). Therefore, in-depth understanding of the regulatory mechanisms involved in cholesterol-mediated cancer drug resistance is required, which may provide useful clues to develop strategies to combat this mounting problem.

## SUPPLEMENTARY MATERIALS



## References

[1] Romano G, Veneziano D, Acunzo M, Croce CM (2017). Small non-coding RNA and cancer.. Carcinogenesis.

[2] Moran Y, Agron M, Praher D, Technau U (2017). The evolutionary origin of plant and animal microRNAs. Nat Ecol Evol.

[3] Abue M, Yokoyama M, Shibuya R, Tamai K, Yamaguchi K, Sato I, Tanaka N, Hamada S, Shimosegawa T, Sugamura K, Satoh K (2015). Circulating miR-483-3p and miR-21 is highly expressed in plasma of pancreatic cancer.. Int J Oncol.

[4] Kim VN, Han J, Siomi MC (2009). Biogenesis of small RNAs in animals.. Nat Rev Mol Cell Biol.

[5] Kozomara A, Birgaoanu M, Griffiths-Jones S (2019). miRBase: from microRNA sequences to function.. Nucleic Acids Res.

[6] Lee RC, Feinbaum RL, Ambros V (1993). The C. elegans heterochronic gene lin-4 encodes small RNAs with antisense complementarity to lin-14.. Cell.

[7] Wightman B, Ha I, Ruvkun G (1993). Posttranscriptional regulation of the heterochronic gene lin-14 by lin-4 mediates temporal pattern formation in C. elegans.. Cell.

[8] Pasquinelli AE, Reinhart BJ, Slack F, Martindale MQ, Kuroda MI, Maller B, Hayward DC, Ball EE, Degnan B, Müller P, Spring J, Srinivasan A, Fishman M (2000). Conservation of the sequence and temporal expression of let-7 heterochronic regulatory RNA.. Nature.

[9] Liu H, Lei C, He Q, Pan Z, Xiao D, Tao Y (2018). Nuclear functions of mammalian MicroRNAs in gene regulation, immunity and cancer.. Mol Cancer.

[10] Huang-Doran I, Zhang CY, Vidal-Puig A (2017). Extracellular vesicles: novel mediators of cell communication in metabolic disease.. Trends Endocrinol Metab.

[11] Rupaimoole R, Calin GA, Lopez-Berestein G, Sood AK (2016). miRNA deregulation in cancer cells and the tumor microenvironment.. Cancer Discov.

[12] Rivera-Barahona A, Pérez B, Richard E, Desviat LR (2017). Role of miRNAs in human disease and inborn errors of metabolism.. J Inherit Metab Dis.

[13] Chakraborty C, Sharma AR, Sharma G, Doss CG, Lee SS (2017). Therapeutic miRNA and siRNA: moving from bench to clinic as next generation medicine.. Mol Ther Nucleic Acids.

[14] Wahid F, Shehzad A, Khan T, Kim YY (2010). MicroRNAs: synthesis, mechanism, function, and recent clinical trials.. Biochim Biophys Acta.

[15] Reesink H, Janssen H, Zeuzem S, Lawitz E, Rodriguez-Torres M, Patel K, Chen A, Davis C, King B, Levin A, Hodges MR (2012). 58 Final results = randomized, double-blind, placebo-controlled safety, anti-viral proof-of-concept study of miravirsen, an oligonucleotide targeting mir-122, in treatment-naive patients with genotype 1 chronic hcv infection.. J Hepatol.

[16] van der Ree MH, van der Meer AJ, van Nuenen AC, de Bruijne J, Ottosen S, Janssen HL, Kootstra NA, Reesink HW (2016). Miravirsen dosing in chronic hepatitis C patients results in decreased microRNA-122 levels without affecting other microRNAs in plasma.. Aliment Pharmacol Ther.

[17] Shah MY, Ferrajoli A, Sood AK, Lopez-Berestein G, Calin GA (2016). microRNA Therapeutics in Cancer - An Emerging Concept.. EBioMedicine.

[18] Mihanfar A, Fattahi A, Nejabati HR (2019). MicroRNA-mediated drug resistance in ovarian cancer.. J Cell Physiol.

[19] Tricoli L, Niture S, Chimeh U, Ressom H, Kumar D (2019). Role of microRNAs in the development of hepatocellular carcinoma and drug resistance.. Front Biosci.

[20] Leonetti A, Assaraf YG, Veltsista PD, El Hassouni B, Tiseo M, Giovannetti E (2019). MicroRNAs as a drug resistance mechanism to targeted therapies in EGFR-mutated NSCLC: current implications and future directions.. Drug Resist Updat.

[21] Hata A, Kashima R (2016). Dysregulation of microRNA biogenesis machinery in cancer.. Crit Rev Biochem Mol Biol.

[22] Daugaard I, Hansen TB (2017). Biogenesis and function of ago-associated RNAs.. Trends Genet.

[23] Kwon SC, Nguyen TA, Choi YG, Jo MH, Hohng S, Kim VN, Woo JS (2016). Structure of Human DROSHA.. Cell.

[24] Wilson RC, Tambe A, Kidwell MA, Noland CL, Schneider CP, Doudna JA (2015). Dicer-TRBP complex formation ensures accurate mammalian microRNA biogenesis.. Mol Cell.

[25] Bohnsack MT, Czaplinski K, Gorlich D (2004). Exportin 5 is a RanGTP-dependent dsRNA-binding protein that mediates nuclear export of pre-miRNAs.. RNA.

[26] Romaine SP, Tomaszewski M, Condorelli G, Samani NJ (2015). MicroRNAs in cardiovascular disease: an introduction for clinicians.. Heart.

[27] Broughton JP, Lovci MT, Huang JL, Yeo GW, Pasquinelli AE (2016). Pairing beyond the seed supports microRNA targeting specificity.. Mol Cell.

[28] Griffiths-Jones S, Grocock RJ, van Dongen S, Bateman A, Enright AJ (2006). miRBase: microRNA sequences, targets and gene nomenclature.. Nucleic Acids Res.

[29] Ambros V, Bartel B, Bartel DP, Burge CB, Carrington JC, Chen X, Dreyfuss G, Eddy SR, Griffiths-Jones S, Marshall M, Matzke M, Ruvkun G, Tuschl T (2003). A uniform system for microRNA annotation.. RNA.

[30] Bishop JM (1991). Molecular themes in oncogenesis.. Cell.

[31] Weinberg RA (1991). Tumor suppressor genes.. Science.

[32] Furuno M, Pang KC, Ninomiya N, Fukuda S, Frith MC, Bult C, Kai C, Kawai J, Carninci P, Hayashizaki Y, Mattick JS, Suzuki H (2006). Clusters of internally primed transcripts reveal novel long noncoding RNAs.. PLoS Genet.

[33] Oliveto S, Mancino M, Manfrini N, Biffo S (2017). Role of microRNAs in translation regulation and cancer.. World J Biol Chem.

[34] Acunzo M, Romano G, Wernicke D, Croce CM (2015). MicroRNA and cancer—a brief overview.. Adv Biol Regul.

[35] Calin GA, Dumitru CD, Shimizu M, Bichi R, Zupo S, Noch E, Aldler H, Rattan S, Keating M, Rai K, Rassenti L, Kipps T, Negrini M (2002). Frequent deletions and down-regulation of micro- RNA genes miR15 and miR16 at 13q14 in chronic lymphocytic leukemia.. Proc Natl Acad Sci USA.

[36] Peng Y, Croce CM (2016). The role of MicroRNAs in human cancer.. Signal Transduct Target Ther.

[37] Reddy KB (2015). MicroRNA (miRNA) in cancer.. Cancer Cell Int.

[38] Deb B, Uddin A, Chakraborty S (2018). miRNAs and ovarian cancer: an overview.. J Cell Physiol.

[39] Hata A, Lieberman J (2015). Dysregulation of microRNA biogenesis and gene silencing in cancer.. Sci Signal.

[40] He L, He X, Lim LP, de Stanchina E, Xuan Z, Liang Y, Xue W, Zender L, Magnus J, Ridzon D, Jackson AL, Linsley PS, Chen C (2007). A microRNA component of the p53 tumour suppressor network.. Nature.

[41] Chang TC, Wentzel EA, Kent OA, Ramachandran K, Mullendore M, Lee KH, Feldmann G, Yamakuchi M, Ferlito M, Lowenstein CJ, Arking DE, Beer MA, Maitra A, Mendell JT (2007). Transactivation of miR-34a by p53 broadly influences gene expression and promotes apoptosis.. Mol Cell.

[42] Navarro F, Gutman D, Meire E, Cáceres M, Rigoutsos I, Bentwich Z, Lieberman J (2009). miR-34a contributes to megakaryocytic differentiation of K562 cells independently of p53.. Blood.

[43] Corney DC, Hwang CI, Matoso A, Vogt M, Flesken-Nikitin A, Godwin AK, Kamat AA, Sood AK, Ellenson LH, Hermeking H, Nikitin AY (2010). Frequent downregulation of miR-34 family in human ovarian cancers.. Clin Cancer Res.

[44] Kasinski AL, Slack FJ (2012). miRNA-34 prevents cancer initiation and progression in a therapeutically resistant K-ras and p53-induced mouse model of lung adenocarcinoma.. Cancer Res.

[45] Hu H, Gatti RA (2011). MicroRNAs: new players in the DNA damage response.. J Mol Cell Biol.

[46] Lal A, Pan Y, Navarro F, Dykxhoorn DM, Moreau L, Meire E, Bentwich Z, Lieberman J, Chowdhury D (2009). miR-24-mediated downregulation of H2AX suppresses DNA repair in terminally differentiated blood cells.. Nat Struct Mol Biol.

[47] Rupaimoole R, Wu SY, Pradeep S, Ivan C, Pecot CV, Gharpure KM, Nagaraja AS, Armaiz-Pena GN, McGuire M, Zand B, Dalton HJ, Filant J, Miller JB (2014). Hypoxia-mediated downregulation of miRNA biogenesis promotes tumour progression.. Nat Commun.

[48] Devlin C, Greco S, Martelli F, Ivan M (2011). miR-210: more than a silent player in hypoxia.. IUBMB Life.

[49] Xu T, Zhu Y, Xiong Y, Ge YY, Yun JP, Zhuang SM (2009). MicroRNA-195 suppresses tumorigenicity and regulates G1/S transition of human hepatocellular carcinoma cells.. Hepatology.

[50] Mongroo PS, Rustgi AK (2010). The role of the miR-200 family in epithelial-mesenchymal transition.. Cancer Biol Ther.

[51] Kambach DM, Halim AS, Cauer AG, Sun Q, Tristan CA, Celiku O, Kesarwala AH, Shankavaram U, Batchelor E, Stommel JM (2017). Disabled cell density sensing leads to dysregulated cholesterol synthesis in glioblastoma.. Oncotarget.

[52] Gu L, Saha ST, Thomas J, Kaur M (2019). Targeting cellular cholesterol for anticancer therapy.. FEBS J.

[53] Stine JE, Guo H, Sheng X, Han X, Schointuch MN, Gilliam TP, Gehrig PA, Zhou C, Bae-Jump VL (2016). The HMG-CoA reductase inhibitor, simvastatin, exhibits anti-metastatic and anti-tumorigenic effects in ovarian cancer.. Oncotarget.

[54] Kuzu OF, Noory MA, Robertson GP (2016). The role of cholesterol in cancer.. Cancer Res.

[55] Simigdala N, Gao Q, Pancholi S, Roberg-Larsen H, Zvelebil M, Ribas R, Folkerd E, Thompson A, Bhamra A, Dowsett M, Martin LA (2016). Cholesterol biosynthesis pathway as a novel mechanism of resistance to estrogen deprivation in estrogen receptor-positive breast cancer.. Breast Cancer Res.

[56] Jamnagerwalla J, Howard LE, Allott EH, Vidal AC, Moreira DM, Castro-Santamaria R, Andriole GL, Freeman MR, Freedland SJ (2018). Serum cholesterol and risk of high-grade prostate cancer: results from the REDUCE study.. Prostate Cancer Prostatic Dis.

[57] Martin LJ, Melnichouk O, Huszti E, Connelly PW, Greenberg CV, Minkin S, Boyd NF (2015). Serum lipids, lipoproteins, and risk of breast cancer: a nested case-control study using multiple time points.. J Natl Cancer Inst.

[58] Najafi-Shoushtari SH, Kristo F, Li Y, Shioda T, Cohen DE, Gerszten RE, Näär AM (2010). MicroRNA-33 and the SREBP host genes cooperate to control cholesterol homeostasis.. Science.

[59] Adlakha YK, Khanna S, Singh R, Singh VP, Agrawal A, Saini N (2013). Pro-apoptotic miRNA-128-2 Modulates ABCA1, ABCG1 and RXRα Expression and Cholesterol Homeostasis. Cell Death Dis.

[60] Vickers KC, Landstreet SR, Levin MG, Shoucri BM, Toth CL, Taylor RC, Palmisano BT, Tabet F, Cui HL, Rye KA, Sethupathy P, Remaley AT (2014). MicroRNA-223 coordinates cholesterol homeostasis.. Proc Natl Acad Sci USA.

[61] Chimento A, Casaburi I, Avena P, Trotta F, De Luca A, Rago V, Pezzi V, Sirianni R (2019). Cholesterol and Its Metabolites in Tumor Growth: Therapeutic Potential of Statins in Cancer Treatment. Front Endocrinol (Lausanne).

[62] Borgquist S, Giobbie-Hurder A, Ahern TP, Garber JE, Colleoni M, Láng I, Debled M, Ejlertsen B, von Moos R, Smith I, Coates AS, Goldhirsch A, Rabaglio M (2017). Cholesterol, cholesterol-lowering medication use, and breast cancer outcome in the BIG 1-98 study.. J Clin Oncol.

[63] Lagos-Quintana M, Rauhut R, Yalcin A, Meyer J, Lendeckel W, Tuschl T (2002). Identification of tissue-specific microRNAs from mouse.. Curr Biol.

[64] Iliopoulos D, Drosatos K, Hiyama Y, Goldberg IJ, Zannis VI (2010). MicroRNA-370 controls the expression of microRNA-122 and Cpt1α and affects lipid metabolism.. J Lipid Res.

[65] Iliopoulos D, Drosatos K, Hiyama Y, Goldberg IJ, Zannis VI (2010). MicroRNA-370 controls the expression of microRNA-122 and Cpt1alpha and affects lipid metabolism. J Lipid Res.

[66] Gao W, He HW, Wang ZM, Zhao H, Lian XQ, Wang YS, Zhu J, Yan JJ, Zhang DG, Yang ZJ, Wang LS (2012). Plasma levels of lipometabolism-related miR-122 and miR-370 are increased in patients with hyperlipidemia and associated with coronary artery disease.. Lipids Health Dis.

[67] Li M, Wang Q, Liu SA, Zhang JQ, Ju W, Quan M, Feng SH, Dong JL, Gao P, Cheng J (2015). MicroRNA-185-5p mediates regulation of SREBP2 expression by hepatitis C virus core protein.. World J Gastroenterol.

[68] Wagschal A, Najafi-Shoushtari SH, Wang L, Goedeke L, Sinha S, deLemos AS, Black JC, Ramírez CM, Li Y, Tewhey R, Hatoum I, Shah N, Lu Y (2015). Genome-wide identification of microRNAs regulating cholesterol and triglyceride homeostasis.. Nat Med.

[69] Wang H, Luo J, Zhang T, Tian H, Ma Y, Xu H, Yao D, Loor JJ (2016). MicroRNA-26a/b and their host genes synergistically regulate triacylglycerol synthesis by targeting the INSIG1 gene.. RNA Biol.

[70] Ramirez CM, Dávalos A, Goedeke L, Salerno AG, Warrier N, Cirera-Salinas D, Suárez Y, Fernández-Hernando C (2011). MicroRNA-758 regulates cholesterol efflux through posttranscriptional repression of ATP-binding cassette transporter A1.. Arterioscler Thromb Vasc Biol.

[71] Gottesman MM (2002). Mechanisms of cancer drug resistance.. Annu Rev Med.

[72] Gottesman MM, Lavi O, Hall MD, Gillet JP (2016). Toward a better understanding of the complexity of cancer drug resistance.. Annu Rev Pharmacol Toxicol.

[73] Mansoori B, Mohammadi A, Davudian S, Shirjang S, Baradaran B (2017). The different mechanisms of cancer drug resistance: a brief review.. Adv Pharm Bull.

[74] Hultsch S, Kankainen M, Paavolainen L, Kovanen RM, Ikonen E, Kangaspeska S, Pietiäinen V, Kallioniemi O (2018). Association of tamoxifen resistance and lipid reprogramming in breast cancer.. BMC Cancer.

[75] Lumachi F, Santeufemia DA, Basso SM (2015). Current medical treatment of estrogen receptor-positive breast cancer.. World J Biol Chem.

[76] Tiwary R, Yu W, deGraffenried LA, Sanders BG, Kline K (2011). Targeting cholesterol-rich microdomains to circumvent tamoxifen-resistant breast cancer.. Breast Cancer Res.

[77] Kong Y, Cheng L, Mao F, Zhang Z, Zhang Y, Farah E, Bosler J, Bai Y, Ahmad N, Kuang S, Li L, Liu X (2018). Inhibition of cholesterol biosynthesis overcomes enzalutamide resistance in castration-resistant prostate cancer (CRPC).. J Biol Chem.

[78] Garcia-Bermudez J, Baudrier L, Bayraktar EC, Shen Y, La K, Guarecuco R, Yucel B, Fiore D, Tavora B, Freinkman E, Chan SH, Lewis C, Min W (2019). Squalene accumulation in cholesterol auxotrophic lymphomas prevents oxidative cell death.. Nature.

[79] Yamamoto Y, Tomiyama A, Sasaki N, Yamaguchi H, Shirakihara T, Nakashima K, Kumagai K, Takeuchi S, Toyooka T, Otani N, Wada K, Narita Y, Ichimura K (2018). Intracellular cholesterol level regulates sensitivity of glioblastoma cells against temozolomide-induced cell death by modulation of caspase-8 activation
*via*
death receptor 5-accumulation and activation in the plasma membrane lipid raft.. Biochem Biophys Res Commun.

[80] Weber TG, Osl F, Renner A, Pöschinger T, Galbán S, Rehemtulla A, Scheuer W (2014). Apoptosis imaging for monitoring DR5 antibody accumulation and pharmacodynamics in brain tumors non-invasively.. Cancer Res.

[81] Chaudhary PM, Roninson IB (1993). Induction of multidrug resistance in human cells by transient exposure to different chemotherapeutic drugs.. J Natl Cancer Inst.

[82] Rudas M, Filipits M, Taucher S, Stranzl T, Steger GG, Jakesz R, Pirker R, Pohl G (2003). Expression of MRP1, LRP and Pgp in breast carcinoma patients treated with preoperative chemotherapy.. Breast Cancer Res Treat.

[83] Chevillard S, Pouillart P, Beldjord C, Asselain B, Beuzeboc P, Magdelénat H, Vielh P (1996). Sequential assessment of multidrug resistance phenotype and measurement of S-phase fraction as predictive markers of breast cancer response to neoadjuvant chemotherapy.. Cancer.

[84] Kamau SW, Krämer SD, Günthert M, Wunderli-Allenspach H (2005). Effect of the modulation of the membrane lipid composition on the localization and function of P-glycoprotein in MDR1-MDCK cells.. In Vitro Cell Dev Biol Anim.

[85] Mack JT, Townsend DM, Beljanski V, Tew KD (2007). The ABCA2 transporter: intracellular roles in trafficking and metabolism of LDL-derived cholesterol and sterol-related compounds.. Curr Drug Metab.

[86] Rahgozar S, Moafi A, Abedi M, Entezar-E-Ghaem M, Moshtaghian J, Ghaedi K, Esmaeili A, Montazeri F (2014). mRNA expression profile of multidrug-resistant genes in acute lymphoblastic leukemia of children, a prognostic value for ABCA3 and ABCA2.. Cancer Biol Ther.

[87] Boonstra R, Timmer-Bosscha H, van Echten-Arends J, van der Kolk DM, van den Berg A, de Jong B, Tew KD, Poppema S, de Vries EG (2004). Mitoxantrone resistance in a small cell lung cancer cell line is associated with ABCA2 upregulation.. Br J Cancer.

[88] Montero J, Morales A, Llacuna L, Lluis JM, Terrones O, Basañez G, Antonsson B, Prieto J, García-Ruiz C, Colell A, Fernández-Checa JC (2008). Mitochondrial cholesterol contributes to chemotherapy resistance in hepatocellular carcinoma.. Cancer Res.

[89] Bruchovsky N, Klotz L, Crook J, Goldenberg SL (2007). Locally advanced prostate cancer—biochemical results from a prospective phase II study of intermittent androgen suppression for men with evidence of prostate-specific antigen recurrence after radiotherapy.. Cancer.

[90] Wang Y, Kreisberg JI, Ghosh PM (2007). Cross-talk between the androgen receptor and the phosphatidylinositol 3-kinase/Akt pathway in prostate cancer.. Curr Cancer Drug Targets.

[91] Locke JA, Guns ES, Lubik AA, Adomat HH, Hendy SC, Wood CA, Ettinger SL, Gleave ME, Nelson CC (2008). Androgen levels increase by intratumoral de novo steroidogenesis during progression of castration-resistant prostate cancer.. Cancer Res.

[92] Roy M, Kung HJ, Ghosh PM (2011). Statins and prostate cancer: role of cholesterol inhibition
*vs*
prevention of small GTP-binding proteins.. Am J Cancer Res.

[93] Mokarram P, Alizadeh J, Razban V, Barazeh M, Solomon C, Kavousipour S (2017). Interconnection of estrogen/testosterone metabolism and mevalonate pathway in breast and prostate cancers.. Curr Mol Pharmacol.

[94] Li YC, Park MJ, Ye SK, Kim CW, Kim YN (2006). Elevated levels of cholesterol-rich lipid rafts in cancer cells are correlated with apoptosis sensitivity induced by cholesterol-depleting agents.. Am J Pathol.

[95] Wang J, Yang M, Li Y, Han B (2015). The role of microRNAs in the chemoresistance of breast cancer.. Drug Dev Res.

[96] Shibayama Y, Kondo T, Ohya H, Fujisawa S, Teshima T, Iseki K (2015). Upregulation of microRNA-126-5p is associated with drug resistance to cytarabine and poor prognosis in AML patients.. Oncol Rep.

[97] Niu J, Xue A, Chi Y, Xue J, Wang W, Zhao Z, Fan M, Yang CH, Shao ZM, Pfeffer LM, Wu J, Wu ZH (2016). Induction of miRNA-181a by genotoxic treatments promotes chemotherapeutic resistance and metastasis in breast cancer.. Oncogene.

[98] Vilquin P, Donini CF, Villedieu M, Grisard E, Corbo L, Bachelot T, Vendrell JA, Cohen PA (2015). MicroRNA-125b upregulation confers aromatase inhibitor resistance and is a novel marker of poor prognosis in breast cancer.. Breast Cancer Res.

[99] Jian J, Xuan F, Qin F, Huang R (2015). Bauhinia championii flavone inhibits apoptosis and autophagy
*via*
the PI3K/Akt pathway in myocardial ischemia/reperfusion injury in rats.. Drug Des Devel Ther.

[100] Zhang Y, Duan G, Feng S (2015). MicroRNA-301a modulates doxorubicin resistance in osteosarcoma cells by targeting AMP-activated protein kinase alpha 1.. Biochem Biophys Res Commun.

[101] Jiao X, Zhao L, Ma M, Bai X, He M, Yan Y, Wang Y, Chen Q, Zhao X, Zhou M, Cui Z, Zheng Z, Wang E, Wei M (2013). MiR-181a enhances drug sensitivity in mitoxantone-resistant breast cancer cells by targeting breast cancer resistance protein (BCRP/ABCG2).. Breast Cancer Res Treat.

[102] Nakanishi T, Ross DD (2012). Breast cancer resistance protein (BCRP/ABCG2): its role in multidrug resistance and regulation of its gene expression.. Chin J Cancer.

[103] Storch CH, Ehehalt R, Haefeli WE, Weiss J (2007). Localization of the human breast cancer resistance protein (BCRP/ABCG2) in lipid rafts/caveolae and modulation of its activity by cholesterol
*in vitro.*. J Pharmacol Exp Ther.

[104] Singh R, Yadav V, Kumar S, Saini N (2015). MicroRNA-195 inhibits proliferation, invasion and metastasis in breast cancer cells by targeting FASN, HMGCR, ACACA and CYP27B1.. Sci Rep.

[105] Li X, Chen YT, Josson S, Mukhopadhyay NK, Kim J, Freeman MR, Huang WC (2013). MicroRNA-185 and 342 inhibit tumorigenicity and induce apoptosis through blockade of the SREBP metabolic pathway in prostate cancer cells.. PLoS One.

[106] DiMarco DM, Fernandez ML (2015). The regulation of reverse cholesterol transport and cellular cholesterol homeostasis by microRNAs.. Biology (Basel).

[107] Tao Z, Shi A, Lu C, Song T, Zhang Z, Zhao J (2015). Breast cancer: epidemiology and etiology.. Cell Biochem Biophys.

[108] Zhou G, Xia J (2018). OmicsNet: a web-based tool for creation and visual analysis of biological networks in 3D space.. Nucleic Acids Res.

[109] Goldstein JL, Brown MS (2015). A century of cholesterol and coronaries: from plaques to genes to statins.. Cell.

[110] Gallagher EJ, Zelenko Z, Neel BA, Antoniou IM, Rajan L, Kase N, LeRoith D (2017). Elevated tumor LDLR expression accelerates LDL cholesterol-mediated breast cancer growth in mouse models of hyperlipidemia.. Oncogene.

[111] Wegner MS, Gruber L, Mattjus P, Geisslinger G, Grösch S (2018). The UDP-glucose ceramide glycosyltransferase (UGCG) and the link to multidrug resistance protein 1 (MDR1).. BMC Cancer.

[112] Kopecka J, Campia I, Olivero P, Pescarmona G, Ghigo D, Bosia A, Riganti C (2011). A LDL-masked liposomal-doxorubicin reverses drug resistance in human cancer cells.. J Control Release.

[113] Yuan J, Yin Z, Tao K, Wang G, Gao J (2018). Function of insulin-like growth factor 1 receptor in cancer resistance to chemotherapy.. Oncol Lett.

[114] Ochnik AM, Baxter RC (2016). Combination therapy approaches to target insulin-like growth factor receptor signaling in breast cancer.. Endocr Relat Cancer.

[115] Sui P, Cao H, Meng L, Hu P, Ma H, Du J (2014). The synergistic effect of humanized monoclonal antibodies targeting insulin-like growth factor 1 receptor (IGF-1R) and chemotherapy.. Curr Drug Targets.

[116] de Groot S, Charehbili A, van Laarhoven HW, Mooyaart AL, Dekker-Ensink NG, van de Ven S, Janssen LG, Swen JJ, Smit VT, Heijns JB, Kessels LW, van der Straaten T, Böhringer S, Dutch Breast Cancer Research Group (2016). Insulin-like growth factor 1 receptor expression and IGF1R 3129G > T polymorphism are associated with response to neoadjuvant chemotherapy in breast cancer patients: results from the NEOZOTAC trial (BOOG 2010-01).. Breast Cancer Res.

[117] Bhasker CR, Friedmann T (2008). Insulin-like growth factor-1 coordinately induces the expression of fatty acid and cholesterol biosynthetic genes in murine C2C12 myoblasts.. BMC Genomics.

[118] Tian Z, Yao G, Song H, Zhou Y, Geng J (2014). IGF2R expression is associated with the chemotherapy response and prognosis of patients with advanced NSCLC.. Cell Physiol Biochem.

[119] Zhao Z, Li Y, Jain A, Chen Z, Liu H, Jin W, Cheng K (2018). Development of a peptide-modified siRNA nanocomplex for hepatic stellate cells.. Nanomedicine (Lond).

[120] Tang Z, Li C, Kang B, Gao G, Li C, Zhang Z (2017). GEPIA: a web server for cancer and normal gene expression profiling and interactive analyses.. Nucleic Acids Res.

[121] Liu YY, Patwardhan GA, Xie P, Gu X, Giuliano AE, Cabot MC (2011). Glucosylceramide synthase, a factor in modulating drug resistance, is overexpressed in metastatic breast carcinoma.. Int J Oncol.

[122] Hartog H, Boezen HM, de Jong MM, Schaapveld M, Wesseling J, van der Graaf WT (2013). Prognostic value of insulin-like growth factor 1 and insulin-like growth factor binding protein 3 blood levels in breast cancer.. Breast.

[123] Benelli R, Venè R, Ferrari N (2018). Prostaglandin-endoperoxide synthase 2 (cyclooxygenase-2), a complex target for colorectal cancer prevention and therapy.. Transl Res.

[124] Li J, Kong X, Zhang J, Luo Q, Li X, Fang L (2013). MiRNA-26b inhibits proliferation by targeting PTGS2 in breast cancer.. Cancer Cell Int.

[125] Costa R, Shah AN, Santa-Maria CA, Cruz MR, Mahalingam D, Carneiro BA, Chae YK, Cristofanilli M, Gradishar WJ, Giles FJ (2017). Targeting Epidermal Growth Factor Receptor in triple negative breast cancer: new discoveries and practical insights for drug development.. Cancer Treat Rev.

[126] Hansen SN, Westergaard D, Thomsen MB, Vistesen M, Do KN, Fogh L, Belling KC, Wang J, Yang H, Gupta R, Ditzel HJ, Moreira J, Brünner N (2015). Acquisition of docetaxel resistance in breast cancer cells reveals upregulation of ABCB1 expression as a key mediator of resistance accompanied by discrete upregulation of other specific genes and pathways.. Tumour Biol.

[127] Heo TH, Wahler J, Suh N (2016). Potential therapeutic implications of IL-6/IL-6R/gp130-targeting agents in breast cancer.. Oncotarget.

[128] Madden SF, Clarke C, Gaule P, Aherne ST, O’Donovan N, Clynes M, Crown J, Gallagher WM (2013). BreastMark: an integrated approach to mining publicly available transcriptomic datasets relating to breast cancer outcome.. Breast Cancer Res.

[129] Adlakha YK, Khanna S, Singh R, Singh VP, Agrawal A, Saini N (2013). Pro-apoptotic miRNA-128-2 modulates ABCA1, ABCG1 and RXRα expression and cholesterol homeostasis.. Cell Death Dis.

[130] Karashchuk G, Karashchuk N, Caksa S, Smith TS, Brodsky AS (2017). Cholesterol pathway determines ovarian cancer drug resistance through transcription factor SREBP2.

[131] Zheng L, Li L, Lu Y, Jiang F, Yang XA (2018). SREBP2 contributes to cisplatin resistance in ovarian cancer cells.. Exp Biol Med (Maywood).

[132] Adlakha Y, Saini N. (2013). miR-128 exerts pro-apoptotic effect in a p53 transcription-dependent and-independent manner via PUMA-Bak axis.. Cell Death Dis.

[133] Hu J, Cheng Y, Li Y, Jin Z, Pan Y, Liu G, Fu S, Zhang Y, Feng K, Feng Y (2014). microRNA-128 plays a critical role in human non-small cell lung cancer tumourigenesis, angiogenesis and lymphangiogenesis by directly targeting vascular endothelial growth factor-C.. Eur J Cancer.

[134] Pan J, Zhou C, Zhao X, He J, Tian H, Shen W, Han Y, Chen J, Fang S, Meng X, Jin X, Gong Z (2018). A two-miRNA signature (miR-33a-5p and miR-128-3p) in whole blood as potential biomarker for early diagnosis of lung cancer.. Sci Rep.

[135] Cai J, Fang L, Huang Y, Li R, Xu X, Hu Z, Zhang L, Yang Y, Zhu X, Zhang H, Wu J, Huang Y, Li J (2017). Simultaneous overactivation of Wnt/β-catenin and TGFβ signalling by miR-128-3p confers chemoresistance-associated metastasis in NSCLC.. Nat Commun.

[136] Zhu Y, Yu F, Jiao Y, Feng J, Tang W, Yao H, Gong C, Chen J, Su F, Zhang Y, Song E (2011). Reduced miR-128 in breast tumor-initiating cells induces chemotherapeutic resistance via Bmi-1 and ABCC5.. Clin Cancer Res.

[137] Yang J, Maika S, Craddock L, King JA, Liu ZM (2008). Chronic activation of AMP-activated protein kinase-alpha1 in liver leads to decreased adiposity in mice.. Biochem Biophys Res Commun.

[138] Moon YA (2017). The SCAP/SREBP pathway: A mediator of hepatic steatosis.. Endocrinol Metab (Seoul).

[139] Dong XY, Tang SQ, Chen JD (2012). Dual functions of Insig proteins in cholesterol homeostasis.. Lipids Health Dis.

[140] Sun S, Zhang G, Sun Q, Wu Z, Shi W, Yang B, Li Y (2016). Insulin-induced gene 2 expression correlates with colorectal cancer metastasis and disease outcome.. IUBMB Life.

[141] Kayashima T, Nakata K, Ohuchida K, Ueda J, Shirahane K, Fujita H, Cui L, Mizumoto K, Tanaka M (2011). Insig2 is overexpressed in pancreatic cancer and its expression is induced by hypoxia.. Cancer Sci.

[142] Novak A, Binnington B, Ngan B, Chadwick K, Fleshner N, Lingwood CA (2013). Cholesterol masks membrane glycosphingolipid tumor-associated antigens to reduce their immunodetection in human cancer biopsies.. Glycobiology.

[143] Beloribi-Djefaflia S, Vasseur S, Guillaumond F (2016). Lipid metabolic reprogramming in cancer cells.. Oncogenesis.

[144] Glaros EN, Kim WS, Quinn CM, Wong J, Gelissen I, Jessup W, Garner B (2005). Glycosphingolipid accumulation inhibits cholesterol efflux
*via*
the ABCA1/apolipoprotein A-I pathway: 1-phenyl-2-decanoylamino-3-morpholino-1-propanol is a novel cholesterol efflux accelerator.. J Biol Chem.

[145] Willeit P, Skroblin P, Kiechl S, Fernández-Hernando C, Mayr M (2016). Liver microRNAs: potential mediators and biomarkers for metabolic and cardiovascular disease?. Eur Heart J.

[146] Lv J, Fu Z, Shi M, Xia K, Ji C, Xu P, Lv M, Pan B, Dai L, Xie H (2015). Systematic analysis of gene expression pattern in has-miR-760 overexpressed resistance of the MCF-7 human breast cancer cell to doxorubicin.. Biomed Pharmacother.

[147] Prochazka L, Koudelka S, Dong LF, Stursa J, Goodwin J, Neca J, Slavik J, Ciganek M, Masek J, Kluckova K, Nguyen M, Turanek J, Neuzil J (2013). Mitochondrial targeting overcomes ABCA1-dependent resistance of lung carcinoma to α-tocopheryl succinate.. Apoptosis.

[148] Wegner MS, Schömel N, Gruber L, Örtel SB, Kjellberg MA, Mattjus P, Kurz J, Trautmann S, Peng B, Wegner M, Kaulich M, Ahrends R, Geisslinger G, Grösch S (2018). UDP-glucose ceramide glucosyltransferase activates AKT, promoted proliferation, and doxorubicin resistance in breast cancer cells.. Cell Mol Life Sci.

[149] Masciarelli S, Fontemaggi G, Di Agostino S, Donzelli S, Carcarino E, Strano S, Blandino G (2014). Gain-of-function mutant p53 downregulates miR-223 contributing to chemoresistance of cultured tumor cells.. Oncogene.

[150] Rayner KJ, Suárez Y, Dávalos A, Parathath S, Fitzgerald ML, Tamehiro N, Fisher EA, Moore KJ, Fernández-Hernando C (2010). MiR-33 contributes to the regulation of cholesterol homeostasis. Science.

[151] Gong B, Liu WW, Nie WJ, Li DF, Xie ZJ, Liu C, Liu YH, Mei P, Li ZJ (2015). MiR-21/RASA1 axis affects malignancy of colon cancer cells
*via*
RAS pathways.. World J Gastroenterol.

[152] Choi PW, Ng SW (2017). The functions of MicroRNA-200 family in ovarian cancer: beyond epithelial-mesenchymal transition.. Int J Mol Sci.

[153] Wang HY, Tu YS, Long J, Zhang HQ, Qi CL, Xie XB, Li SH, Zhang YJ (2015). SRF-miR‑29b-MMP2 axis inhibits NSCLC invasion and metastasis.. Int J Oncol.

[154] Ma X, Yang X, Bao W, Li S, Liang S, Sun Y, Zhao Y, Wang J, Zhao C (2018). Circular RNA circMAN2B2 facilitates lung cancer cell proliferation and invasion
*via*
miR-1275/FOXK1 axis.. Biochem Biophys Res Commun.

[155] Vychytilova-Faltejskova P, Kiss I, Klusova S, Hlavsa J, Prochazka V, Kala Z, Mazanec J, Hausnerova J, Kren L, Hermanova M, Lenz J, Karasek P, Vyzula R, Slaby O (2015). MiR-21, miR-34a, miR-198 and miR-217 as diagnostic and prognostic biomarkers for chronic pancreatitis and pancreatic ductal adenocarcinoma.. Diagn Pathol.

[156] Zhao Y, Zhao L, Ischenko I, Bao Q, Schwarz B, Nieß H, Wang Y, Renner A, Mysliwietz J, Jauch KW, Nelson PJ, Ellwart JW, Bruns CJ, Camaj P (2015). Antisense inhibition of microRNA-21 and microRNA-221 in tumor-initiating stem-like cells modulates tumorigenesis, metastasis, and chemotherapy resistance in pancreatic cancer.. Target Oncol.

[157] Bahena-Ocampo I, Espinosa M, Ceballos-Cancino G, Lizarraga F, Campos-Arroyo D, Schwarz A, Maldonado V, Melendez-Zajgla J, Garcia‐Lopez P (2016). miR-10b expression in breast cancer stem cells supports self-renewal through negative PTEN regulation and sustained AKT activation.. EMBO Rep.

[158] Eades G, Wolfson B, Zhang Y, Li Q, Yao Y, Zhou Q (2015). lincRNA-RoR and miR-145 regulate invasion in triple-negative breast cancer
*via*
targeting ARF6.. Mol Cancer Res.

[159] Yang Q, Wang Y, Lu X, Zhao Z, Zhu L, Chen S, Wu Q, Chen C, Wang Z (2015). MiR-125b regulates epithelial-mesenchymal transition
*via*
targeting Sema4C in paclitaxel-resistant breast cancer cells.. Oncotarget.

[160] Kim J, Yoon H, Ramírez CM, Lee SM, Hoe HS, Fernández-Hernando C, Kim J (2012). MiR-106b impairs cholesterol efflux and increases Aβ levels by repressing ABCA1 expression.. Exp Neurol.

[161] Wang PY, Li YJ, Zhang S, Li ZL, Yue Z, Xie N, Xie SY (2010). Regulating A549 cells growth by ASO inhibiting miRNA expression.. Mol Cell Biochem.

[162] Garofalo M, Jeon YJ, Nuovo GJ, Middleton J, Secchiero P, Joshi P, Alder H, Nazaryan N, Di Leva G, Romano G, Crawford M, Nana-Sinkam P, Croce CM (2015). Correction: MiR-34a/c-dependent PDGFR-α/β downregulation inhibits tumorigenesis and enhances TRAIL-induced apoptosis in lung cancer.. PLoS One.

[163] Lee J, Padhye A, Sharma A, Song G, Miao J, Mo YY, Wang L, Kemper JK (2010). A pathway involving farnesoid X receptor and small heterodimer partner positively regulates hepatic sirtuin 1 levels
*via*
microRNA-34a inhibition.. J Biol Chem.

[164] Esau C, Davis S, Murray SF, Yu XX, Pandey SK, Pear M, Watts L, Booten SL, Graham M, McKay R, Subramaniam A, Propp S, Lollo BA (2006). miR-122 regulation of lipid metabolism revealed by
*in vivo*
antisense targeting.. Cell Metab.

[165] Xu Y, Xia F, Ma L, Shan J, Shen J, Yang Z, Liu J, Cui Y, Bian X, Bie P, Qian C (2011). MicroRNA-122 sensitizes HCC cancer cells to adriamycin and vincristine through modulating expression of MDR and inducing cell cycle arrest.. Cancer Lett.

[166] Yang T, Zheng ZM, Li XN, Li ZF, Wang Y, Geng YF, Bai L, Zhang XB (2013). MiR-223 modulates multidrug resistance
*via*
downregulation of ABCB1 in hepatocellular carcinoma cells.. Exp Biol Med (Maywood).

[167] Zhou Y, Huang Z, Wu S, Zang X, Liu M, Shi J (2014). miR-33a is up-regulated in chemoresistant osteosarcoma and promotes osteosarcoma cell resistance to cisplatin by down-regulating TWIST.. J Exp Clin Cancer Res.

[168] Lee YS, Lee HH, Park J, Yoo EJ, Glackin CA, Choi YI, Jeon SH, Seong RH, Park SD, Kim JB (2003). Twist2, a novel ADD1/SREBP1c interacting protein, represses the transcriptional activity of ADD1/SREBP1c.. Nucleic Acids Res.

