# Supplementary material for: microRNAs targeting cellular cholesterol: implications for combating anticancer drug resistance
Source: Genes Cancer. 2020;11(1-2):20–42. doi: 10.18632/genesandcancer.202 (PMC7289906; doi:10.18632/genesandcancer.202)
Supplement: Supplementary file 1 [file ganc-11-20-s001.pdf]

microRNAs targeting cellular cholesterol: implications for combating anticancer drug resistance – Monchusi et al

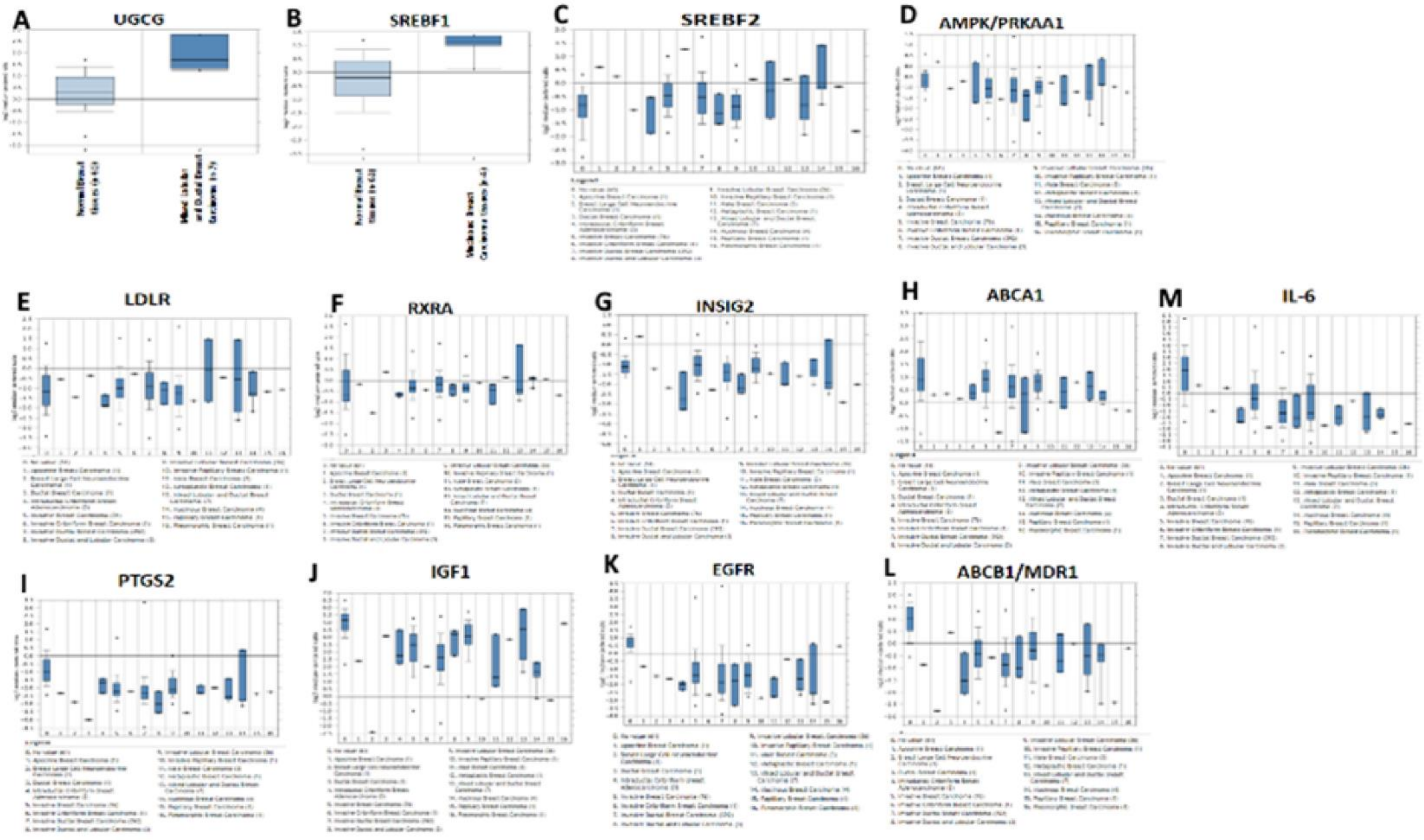

Supplementary Figure 1: Box charts showing the association between mRNA expression of genes (A-M) regulated by hsa-miR-128 and hsa-miR-223 and progression in breast tumour samples versus normal breast samples analysed by Oncomine database (https://www.oncomine.org/resource/main.html) using the TCGA dataset. No disease/Normal breast samples are presented by light blue boxplots while disease/breast tumour tissue samples in dark blue boxplots. Dataset were queried by using these settings: overexpression fold change - top 10%, a threshold at p value = 0.01 and fold change = 2.

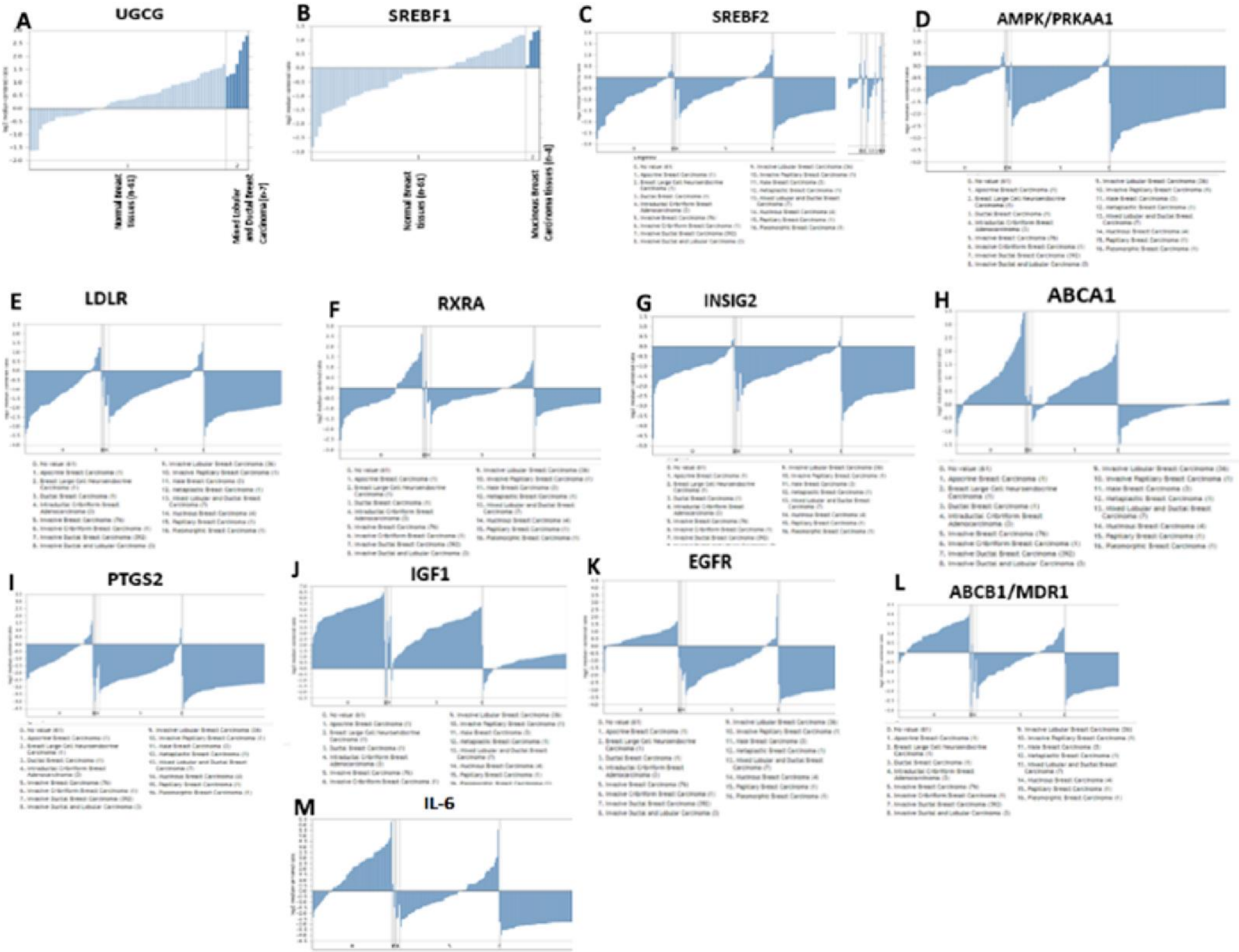

Supplementary Figure 2: Bar charts showing the association between mRNA expression of genes (A-M) regulated by hsa-miR-128 and hsa-miR-223 and progression in breast tumours samples versus normal breast samples analysed by Oncomine database (https://www.oncomine.org/resource/main.html) using the TCGA dataset. No disease/Normal breast samples are presented by light blue bar plots while disease/breast tumour tissue samples in dark blue bar plots. Dataset were queried by using these settings: overexpression fold change - top 10%, a threshold at p value = 0.01 and fold change = 2.
